# Single transcriptional and translational preQ_1_ riboswitches adopt similar pre-folded ensembles that follow distinct folding pathways into the same ligand-bound structure

**DOI:** 10.1093/nar/gkt798

**Published:** 2013-09-03

**Authors:** Krishna C. Suddala, Arlie J. Rinaldi, Jun Feng, Anthony M. Mustoe, Catherine D. Eichhorn, Joseph A. Liberman, Joseph E. Wedekind, Hashim M. Al-Hashimi, Charles L. Brooks, Nils G. Walter

**Affiliations:** ^1^Biophysics, University of Michigan, Ann Arbor, MI 48109, USA, ^2^Single Molecule Analysis Group, University of Michigan, Ann Arbor, MI 48109, USA, ^3^Department of Chemistry, University of Michigan, Ann Arbor, MI 48109, USA, ^4^Program in Chemical Biology, University of Michigan, Ann Arbor, MI 48109, USA, ^5^Department of Biochemistry and Biophysics, Center for RNA Biology, University of Rochester School of Medicine and Dentistry, Rochester, NY 14642, USA and ^6^Center for Theoretical Biological Physics, University of California San Diego, San Diego, CA 92037, USA

## Abstract

Riboswitches are structural elements in the 5′ untranslated regions of many bacterial messenger RNAs that regulate gene expression in response to changing metabolite concentrations by inhibition of either transcription or translation initiation. The preQ_1_ (7-aminomethyl-7-deazaguanine) riboswitch family comprises some of the smallest metabolite sensing RNAs found in nature. Once ligand-bound, the transcriptional *Bacillus subtilis* and translational *Thermoanaerobacter tengcongensis* preQ_1_ riboswitch aptamers are structurally similar RNA pseudoknots; yet, prior structural studies have characterized their ligand-free conformations as largely unfolded and folded, respectively. In contrast, through single molecule observation, we now show that, at near-physiological Mg^2+^ concentration and pH, both ligand-free aptamers adopt similar pre-folded state ensembles that differ in their ligand-mediated folding. Structure-based Gō-model simulations of the two aptamers suggest that the ligand binds late (*Bacillus subtilis*) and early (*Thermoanaerobacter tengcongensis*) relative to pseudoknot folding, leading to the proposal that the principal distinction between the two riboswitches lies in their relative tendencies to fold via mechanisms of conformational selection and induced fit, respectively. These mechanistic insights are put to the test by rationally designing a single nucleotide swap distal from the ligand binding pocket that we find to predictably control the aptamers′ pre-folded states and their ligand binding affinities.

## INTRODUCTION

Riboswitches are highly structured non-coding RNA motifs that are found in up to 4% of all 5′-untranslated regions of bacterial messenger RNAs and respond to cellular metabolites or other signals to control gene expression (1–5). Riboswitches are composed of a highly conserved aptamer domain, which binds a ligand, and a downstream variable expression platform that modulates the genetic on/off switch. Regulation of gene expression is achieved through one of multiple possible modes, most commonly transcription attenuation and inhibition of translation initiation. Although the general principles of genetic regulation by riboswitches are understood, the molecular basis of their action remains largely elusive.

**Figure 1.**
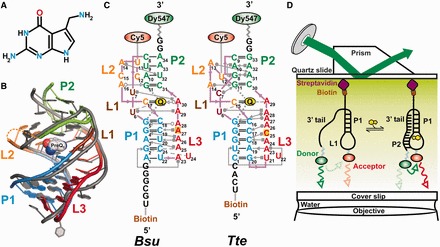
Structural comparison of the *Bsu* and *Tte* preQ_1_ riboswitches. (**A**) Structure of preQ_1_ (7-aminomethyl-7-deazaguanine). (**B**) Structural overlay of the *Bsu* (colored, PDB ID 3FU2, chain A) and *Tte* (gray, PDB ID 3Q50) riboswitch crystal structures. The sugar-phosphate backbone is shown as a single ribbon. preQ_1_ is space-filled and colored as in A. Secondary structure elements are color-coded as indicated. (**C**) Secondary structure maps of the *Bsu* and *Tte* riboswitches with interactions shown in Leontis–Westhof nomenclature (14). Individual secondary structures are color-coded as in (B), and the locations of fluorophores and biotin are indicated. (**D**) Prism-based TIRFM setup for smFRET.

Riboswitches respond to a variety of ligands including nucleobases (6,7), amino acids (8,9), cofactors of metabolic enzymes (10,11) and metal ions (12,13) and are found in a multitude of bacterial species (5). The 7-Aminomethyl-7-deazaguanine, or preQ_1_, is one such ligand (Figure 1A). It is derived from guanine and is an intermediate in the queuosine biosynthetic pathway in bacteria (15,16). Queuosine is found in bacteria and eukaryotes at the wobble position of transfer RNAs for His, Tyr, Asn and Asp (17), where it is thought to be essential for translational fidelity (18–20) as well as bacterial virulence (21). The family of preQ_1_ riboswitches encompasses some of the smallest ligand-responsive RNAs found in nature, making them an ideal target for mechanistic studies. The *Bacillus subtilis* (*Bsu*) and related preQ_1_ riboswitch aptamers in particular have been studied extensively (22–31) and are known to regulate the *queCDEF* operon through transcription termination (32). In contrast, the preQ_1_ riboswitch aptamer from *Thermoanaerobacter tengcongensis* (*Tte*) controls the expression of a putative preQ_1_ transporter and has been implicated as translationally operating wherein ligand binding sequesters the first two nucleotides of the Shine–Dalgarno sequence through Watson–Crick base pairing (33,34).

In the *Bsu* riboswitch, the aptamer has to bind the ligand and stably fold into the pseudoknot structure to switch off gene expression before the competing anti-terminator hairpin is formed in the expression platform, whereas in the *Tte* riboswitch, the expression platform partially overlaps with the ligand-binding aptamer domain. In either case, ligand binding and folding of the aptamer domain is a key event in the regulation of gene expression that is not well understood. Crystal structures of the preQ_1_ bound *Bsu* (22) and *Tte* (34) aptamer domains (referred to henceforth simply as ‘riboswitches’) overlay closely with a backbone unit-vector RMSD (using an all-C3′ atom trace) of only 1.8 Å, as based on the RNA structure alignment program SARA (35) (Figure 1B). This value is comparable with a unit-vector RMSD of 1.6 Å between the lowest-energy nuclear magnetic resonance (NMR) (26) and crystal (22) structures of the *Bsu* riboswitch. Both riboswitches adopt classic H-type pseudoknot structures containing a 5-bp stem P1, a 2-nt loop L1, followed by a 4-bp stem P2 and loops L2 and L3 (Figure 1C). In both riboswitches, the last nucleotide of L2 is a cytidine that recognizes preQ_1_ through Watson–Crick base pairing (Figure 1C). There are only a few relatively subtle differences between the riboswitches (Figure 1C). For example, the P2 stem of the *Bsu* riboswitch bears three Watson–Crick and a single non-canonical C8-A34 bp, whereas the *Tte* riboswitch has two Watson–Crick and two non-canonical bp, G8-A31 and A10-A32. In addition, the L2 loops in the *Bsu* and *Tte* riboswitches are 6- and 4-nt in length, respectively, and the 6-adenosine containing L3 loop in the *Bsu* riboswitch is interrupted by a C insertion in the *Tte* riboswitch (14).

Despite their structural similarities in the ligand bound state, discrepancies between the conformational behaviors of these two riboswitches have been reported for the ligand-free form. NMR studies of the *Bsu* riboswitch (26,36) have suggested a largely unfolded conformation in which its 3′ tail (encompassing L3 and the 3′ segment of P2, Figure 1C) does not form tertiary interactions at 27°C. Such an extended conformation lacks a pre-organized binding site, leaving unresolved the question of how this ‘open’ conformation senses ligand. In addition, a transcriptionally acting riboswitch must bind ligand efficiently during the narrow time window of ∼1-2 s during which RNA polymerase proceeds from the 3′ end of the aptamer domain to the 3′ end of the intrinsic terminator hairpin and, therefore, an unfolded ligand-free conformation would appear ill-suited for the *Bsu* riboswitch. In contrast, X-ray crystallography and small angle X-ray scattering (SAXS) studies on the *Tte* riboswitch (34) suggest that it does form a pre-folded tertiary conformation poised for recognition in the absence of ligand. Such pre-folding of the *Tte* riboswitch at room temperature may not be surprising, given its origin from a thermophilic bacterium that grows optimally at 75°C where the riboswitch may be less folded. The contrasting behavior reported so far for these two structurally similar riboswitches prompted us to investigate their conformational distributions and dynamics under comparable, physiologically relevant buffer conditions. In addition, even though some structural data are available, how the two riboswitches compare in their transition from the ligand-free to the ligand-bound form to affect gene expression is still unclear.

Here, we combine single molecule fluorescence resonance energy transfer (smFRET) (37–39) with computational techniques to compare the folding behavior of the *Bsu* and *Tte* riboswitches. We show that in the absence of ligand, both the *Bsu* and *Tte* riboswitches exist in a similar ensemble of conformations, contrary to previous studies. Both riboswitches exhibit a major population of a ‘pre-folded’ state ensemble wherein their 3′ tail adopts transient interactions with the P1-L1 stem-loop, reminiscent of the fully folded state, and a minor population of a folded-like state. The pre-folded state is poised to bind ligand and, for the *Tte* riboswitch, we find evidence that it can sense preQ_1_. Subtle differences exist in the folding behavior of the transcriptional and translational preQ_1_ riboswitches that can in part be attributed to differential local flexibility of the single-stranded A-rich 3′ tail (36), which leads to differences in long-range transient interactions. Together with results from structure-based Gō-model folding simulations, we show that these differences lead to distinct folding pathways for the transcriptional and translational riboswitches that can be classified as biased toward late ligand binding to an already preformed RNA pocket (conformational selection) and early ligand binding followed by the binding pocket folding around it (induced fit), respectively. Our study unveils design principles for pseudoknot folding that are dependent on the dynamic properties of the single-stranded 3′ tail, a notion we put to the test by rationally reengineering the riboswitches’ pre-folded states and ligand binding affinities through point mutations in this tail. Our results highlight the benefits of comparative studies, establish a framework for delineating conformational selection and induced fit pathways and contribute to an emerging view that environmental conditions as well as distal sequence variations fine-tune the ligand binding properties of riboswitches and RNA in general.

## MATERIALS AND METHODS

### Preparation of RNAs for smFRET

All RNA constructs were synthesized by Dharmacon Inc. (Fayette, CO) with 5′ biotin modification, 3′ DY547 label and 5-aminoallyl-uridine (5 NU) label at position U12 (*Tte*) and U13 (*Bsu*) for later functionalization with Cy5 (Figure 1). Oligonucleotides were deprotected following the manufacturer’s instructions. For labeling each construct, one dye pack of the Cy5-NHS ester (GE Healthcare) was dissolved in 30 µl of DMSO and used to label ∼3.4 nmol RNA in a total reaction volume of 50 µl containing 0.1 M sodium bicarbonate buffer (pH 8.7). The reactions were incubated and tumbled at room temperature in the dark for 4 h. Reaction volumes were adjusted to 500 μl with deionized water and loaded onto a Nap-5 gel filtration column (GE Healthcare) for desalting and removal of excess free dye. Fractions containing the RNA were collected, ethanol precipitated and pellets were resuspended in 50 μl of deionized water.

### Single molecule FRET

We assembled a microfluidic channel on a quartz slide with an inlet and outlet port and coated it with biotinylated-BSA, followed by streptavidin, as previously described (38,40). We folded the RNA by heating at 70°C for 2 min and allowing it to cool to RT for at least 20 min in near-physiological smFRET buffer [50 mM Tris–HCl (pH 7.5), 100 mM KCl, 1 mM MgCl_2_] in the presence or absence of preQ_1_. In all, 100 µl of 10–50 pM of the heat annealed RNA was flowed onto the slide and incubated for 10 min for binding. Excess RNA was removed by flowing 100–200 µl of smFRET buffer with or without preQ_1_ through the channel. An oxygen scavenging system was included in smFRET buffer (+/− preQ_1_), consisting of 5 mM protocatechuic acid and 50 nM protocatechuate-3,4-dioxygenase to slow photobleaching and 2 mM Trolox to reduce photoblinking (39). DY547 was directly excited using a 532 nm laser, and emission from DY547 and Cy5 fluorophores was simultaneously recorded using an intensified CCD camera (I-Pentamax, Princeton Instruments) at 100 ms time resolution (39–44). Experiments at 33 ms data acquisition were performed on a similar prism-based TIRF setup with an EMCCD camera (iXon, Andor Technology). smFRET time traces were extracted from the raw movie files using IDL (Research Systems) and analyzed using Matlab (The Math Works) scripts. Genuine fluorescence traces were selected manually based on the following features: single-step photobleaching, a signal-to-noise ratio of >5:1, a total (donor + acceptor) fluorescence intensity of >300 (arbitrary units) and a total fluorescence duration of >10 s. The FRET ratio was calculated as I_A_/(I_A_ + I_D_), where I_A_ and I_D_ represent the background corrected fluorescence intensities of the acceptor (Cy5) and donor (DY547) fluorophores, respectively. FRET distribution histograms were plotted using OriginLab 8.1. Hidden Markov Modeling (HMM) analysis was performed on smFRET traces using the segmental k-means algorithm in the QuB software suite as described (45). We used a two-state model (with mid-FRET and high-FRET states) to idealize the data; a third zero-FRET state was included to account for a few blinking events. Transition Occupancy Density Plots (TODPs) were then generated from the idealized data using Matlab (45).

### Cross-correlation analysis

Cross-correlation analysis between the donor and acceptor signal intensities of individual smFRET traces was performed similar to previous studies (46) using custom written Matlab programs. Cross-correlation functions were fit with single-exponentials to obtain the reported transition time constants (that are the inverse of the sum of the forward and backward rate constants).

### Isothermal titration calorimetry

The calorimetric methods were conducted as described (47) with minor modifications. *T. tengcongensis* preQ_1_-I riboswitch 33-mer was chemically synthesized (Dharmacon or Fidelity Systems Inc) and purified by reverse phase HPLC (48). The preQ_1_ ligand was prepared by chemical synthesis (LeadGen Labs, LLC). Lyophilized RNA samples were dissolved in 50 mM HEPES-NaOH, pH 7.0, containing 100 mM NaCl and the solution was heated to 65°C for 5 min. MgCl_2_ was added slowly to a final concentration of 6 mM, followed by slow cooling to 24°C. The folded RNA sample was dialyzed overnight at 4°C against 4 L of 100 mM NaCl with 6 mM MgCl_2_ buffered at pH 7.0 by 50 mM HEPES-NaOH, pH 7.0. Following dialysis, riboswitch samples were diluted with dialysis buffer to: 5 µM for the samples analyzed at 25°C, and 3 µM for the samples analyzed at 60°C. PreQ_1_ was dissolved in dialysis buffer to a concentration 7- to 12-fold higher than the RNA. Isothermal titration calorimetry (ITC) measurements were conducted by use of a VP-ITC calorimeter (MicroCal Inc). Titrations were conducted by injecting preQ_1_ from the syringe into the riboswitch using 28 or 29 injections of 10 μl each – except for the first injection of 3 μl; an interval of 260 or 300 sec was used between injections. The resulting thermograms were analyzed with Origin 7.0 (MicroCal) using a 1:1 binding model. Titrations were performed in duplicate at each temperature. Representative titrations and curve fits are shown in Supplementary Figure S5.

### NMR spectroscopy

All NMR experiments were performed at 298 K on an Avance Bruker 600 MHz spectrometer equipped with a triple-resonance cryogenic (5 mm) probe. NMR spectra were analyzed using NMR Draw (49) and Sparky 3. Uniformly labeled ^13^C/^15^N samples were prepared by *in vitro* transcription using T7 RNA polymerase as described previously (50). RNA samples were repeatedly exchanged into NMR buffer (25 mM NaCl, 15 mM Na_i_PO_4_, pH 6.4, 0.1 mM EDTA) using an Ultra-4 Amicon (Millipore Corp.). Final RNA concentrations were 1-2 mM. MgCl_2_ titrations of the *Bsu* aptamer were performed by the incremental addition of MgCl_2_ to a 0.2 mM RNA sample. A 4:1 ratio of preQ_1_ was added to a ∼0.5 mM RNA sample to obtain the preQ_1_-bound sample. Chemical shift differences were determined through 2D ^1^H-^13^C and ^1^H-^15^N HSQC experiments.

### TOPRNA Simulations

TOPological modeling of RNA (TOPRNA) uses three primary pseudo atoms to represent the base (B), sugar (S), and phosphate (P) moieties of an RNA nucleotide. Base pairs are treated as permanent bonds between paired B atoms and regions of contiguous base pairs are parameterized through dihedral potentials to assume standard A-form helical structure. A small filler atom was also placed between each set of paired bases to more accurately reproduce the steric profile of a base pair. Non-base-paired residues were parameterized to maintain RNA-like bond lengths and angles between pseudo-atoms, but were otherwise treated as freely rotatable chains. Both base-paired and non-base-paired residue potential parameters were derived from fits of CHARMM (51) potential functions to structural-database derived statistical potentials. Electrostatic interactions were ignored and, with the exception of a small attractive force between base-paired B atoms meant to simulate intra-helix base stacking, all other non-bonded interactions were solely repulsive in nature. The steric radii of these repulsive interactions were approximated from the minimum dimension of the chemical moiety each pseudo-atom represents. Initial coordinates for both the *Bsu* and *Tte* riboswitches were obtained by equilibrating the initially linear chains of the same sequences as the two RNAs used for smFRET (Figure 1C) with constraints that forced the formation of an A-form helical P1 stem. The base paired residues of P1 were then ‘bonded’ together and additional simulation-dependent dihedral and distance restraints were applied to the 3′ tail and/or residues of the P2 stem, followed by further equilibration (see Supplementary Tables S1 and S2 for restraint details). Temperature replica exchange simulations were then performed in CHARMM (51) with the MMTSB replica exchange server (52) using four temperature windows from 300 K to 400 K for 50 000 exchange cycles so as to achieve exhaustive sampling at each condition. 1000 timesteps of Langevian dynamics with 5 ps^−^^1^ friction coefficient and 0.02 ps integration time step were performed in between each exchange cycle. Neither the fluorophores nor linkers were included in these simulations. Estimates of the inter-fluorophore distances expected in solution were then obtained by measuring the distances sampled over the length of the simulations between the base pseudo-atoms of U13 and G36 and U12 and G35 for the *Bsu* and *Tte* molecules, the sites of fluorophore attachment, respectively.

### Gō-Model RNA Simulations

Gō model RNA simulations were performed essentially as described before (25). The function of the Gō model follows the potential form:

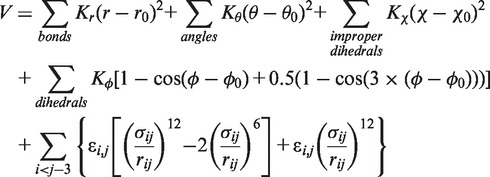

where the equilibrium distances (*r_0_*) and angles (*θ_0_*, *χ_0_* and *φ_0_*) were determined by the native RNA structure. The equilibrium force constants (*K_r_*, *K_θ_*, and *K_χ_*) were adopted from the CHARMM (51) force field based on atom types. Since multiplicity exists in the CHARMM force field for the force constants of dihedrals, we defined *Kφ* as the barrier height from the minimum to the maximum in the CHARMM dihedral potential. It should be noted that, although we are using the native dihedrals in the potential function, the RNA stability is largely dictated by the native contacts as follows. All heavy atoms within 4 Å distance were considered native contact pairs, excluding pairs that are connected within 3-bonds. The native contact potential takes the form of a Lennard-Jones 6–12 potential, where *σ* is the contact distance in the native structure and *ε* is the well depth defining the strength of the native interactions, while all non-native contacts are mutually repulsive with 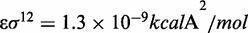. We further partitioned the native contacts into van der Waals contacts and hydrogen bonding contacts. A hydrogen bond was identified when the distance between the acceptor (A) and donor (H-D) was less than 2.4 Å and the A-H-D angle was >120°. For contacts within the RNA, the strength of the interaction *ε* was 0.1 kcal/mol for van der Waals interactions, 2.15 kcal/mol for G-C hydrogen bonds, and 1.58 kcal/mol for all other hydrogen bonds. For contacts between ligand and RNA, *ε* of van der Waals and hydrogen bond interactions were 0.15 and 2.89 kcal/mol, respectively. Each of the 51 folding simulations of the transcriptional and translational riboswitches were performed using the GROMACS simulation package (53) and each simulation was carried out with a different unfolded starting conformation. Stochastic dynamics were performed with a coupling time constant of 1.0 ps and a time step of 2 fs. All bonds were constrained in the simulations.

## RESULTS

### smFRET detects two conformational states in both ligand-free riboswitches with different transition dynamics

To exploit smFRET (37–39) for its ability to elucidate even subtle conformational differences, we chemically modified the crystallized riboswitch sequences by attaching Dy547 as a donor fluorophore at the 3′ terminus and Cy5 as an acceptor fluorophore on a uracil residue of L2 (U13 of *Bsu* and U12 of *Tte*, Figure 1C and Materials and Methods). Both of these uracils were chosen since they are weakly conserved, not involved in any intramolecular interactions, and extrude into solvent (22,26,34). In addition, the donor-acceptor pair is positioned such that pseudoknot formation upon ligand binding is expected to result in close proximity (∼20–30 Å) and thus high FRET, whereas extended or unfolded conformations should result in considerably longer distances and lower FRET (Figure 1D). Finally, we introduced a 5′ biotin to immobilize the RNA on a quartz slide for observation of single molecules by prism-based total internal reflection fluorescence microscopy (TIRFM, Figure 1D), essentially as described (37,39,54), and used a buffer approximating physiological conditions (50 mM Tris-HCl, pH 7.5, 100 mM K^+^, 1 mM Mg^2+^) at room temperature.

**Figure 2.**
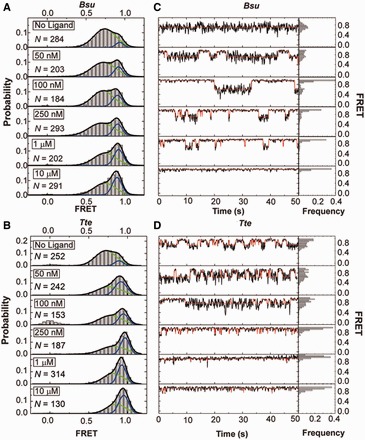
smFRET characterization of single preQ_1_ riboswitch molecules. (**A**) smFRET histograms of the *Bsu* riboswitch with increasing ligand concentration as indicated; *N*, number of molecules sampled. Green and blue lines indicate Gaussian fits of the mid- and high-FRET states, respectively. Black lines indicate cumulative fits. (**B**) Same as in A, but for the *Tte* riboswitch. (**C**) Exemplary FRET time traces of the *Bsu* riboswitch for each condition. Idealized HMM fits are shown as red line. The population of each FRET state is shown as a frequency bar graph to the right. (**D**) Same as in C, but for the *Tte* riboswitch.

In the absence of preQ_1_ ligand, FRET histograms of a few hundred molecules (as indicated in Figure 2A and B), which survey conformational sampling of the entire population (45), exhibit a major broad peak around a FRET value of 0.72 (mean) ± 0.13 (standard deviation, SD) and 0.70 ± 0.13 for the *Bsu* and *Tte* riboswitches, respectively (Figure 2A and B). Additionally, both ligand-free riboswitches contain a minor population of a shorter-distance conformation as indicated by a higher FRET value; the *Bsu* riboswitch shows ∼9% with a FRET value of 0.89 ± 0.05, whereas the *Tte* riboswitch shows ∼11% with a FRET value of 0.90 ± 0.05. Notably, the width (SD) of the 0.7 (or mid)-FRET state is larger than that of the 0.9 (or high)-FRET state, suggesting that the former, in particular, represents a broader dynamic ensemble of structures (or possibly one that our FRET probes more sensitively report on) rather than a single defined conformation. Examination of individual traces shows that the high-FRET state arises in part from relatively short-lived excursions of single molecules up from the mid-FRET state (Figure 2C and D and Supplementary Figures S1–S3). Additionally, some molecules appear to stably persist in the high-FRET state with dwell times of up to tens of seconds (Supplementary Figure S1, panel IV), similar to observations for other riboswitches (55).

A distinction exists when comparing the conformational dynamics of the two riboswitches in the absence of ligand. Almost half of all *Tte* riboswitch molecules undergo dynamic switching between the mid- and high-FRET states, as clearly distinguished at our time resolution (100 ms, Supplementary Figure S1B). In contrast, ∼13% of all *Bsu* riboswitch molecules undergo such observable conformational switching (Supplementary Figure S1A). Closer inspection reveals anti-correlation between the donor and acceptor intensities when conformational switching does occur in either of the two riboswitches, implying true transitions between conformational states of distinct fluorophore distance rather than local quenching effects on just one of the fluorophores. For the *Bsu* riboswitch, the timescales of these transitions are faster than those of the *Tte* riboswitch and are close to our time resolution (Supplementary Figure S1A, 100 ms), suggesting that we may miss a significant number of even faster transitions. We therefore performed cross-correlation analysis (46) on these data, which revealed that an additional 15% of all *Bsu* molecules show anti-correlation between the donor and acceptor signals without transitions revealed by HMM (‘Materials and Methods’ section and Supplementary Figure S4A). To further evaluate the underlying dynamics, we measured the *Bsu* riboswitch in the absence of ligand at 33 ms time resolution. As expected, this faster time resolution increases the population of molecules with HMM-resolved transitions to 64%, with an additional 10% displaying anti-correlation without discernible transitions (Supplementary Figure S4B). Even at this faster time resolution, however, 26% of all *Bsu* molecules in the absence of preQ_1_ reveal no detectable anti-correlation (Supplementary Figure S4B), suggesting that at least this fraction of molecules undergoes transitions that are faster still, similar to observations on the *Fnu* preQ_1_ riboswitch (29).

### Ligand titrations together with coarse-grained simulations identify the two FRET states as pre-folded and folded

A first-order analysis of the mean FRET values associated with the two histogram peaks suggests that the high-FRET state of both riboswitches is consistent with the range of distances expected between fluorophores for the native ligand bound states, which is significantly below the Förster distance of the Dy547-Cy5 fluorophore pair (∼50 Å) (56). The distance of ≤30 Å associated with a FRET value of 0.9 agrees well with the crystal structures of the ligand-bound *Bsu* (22) and *Tte* (33,34) riboswitches, as well as with that of the ligand-free *Tte* riboswitch (34). As discussed earlier in the text, the broad mid-FRET state likely reflects a dynamic ensemble of partially unfolded states. However, its relatively high FRET value of 0.7, corresponding to a donor-acceptor distance of ∼45 Å, appears much higher than that expected if the conformation of the 3′-tail were truly random.

**Figure 3.**
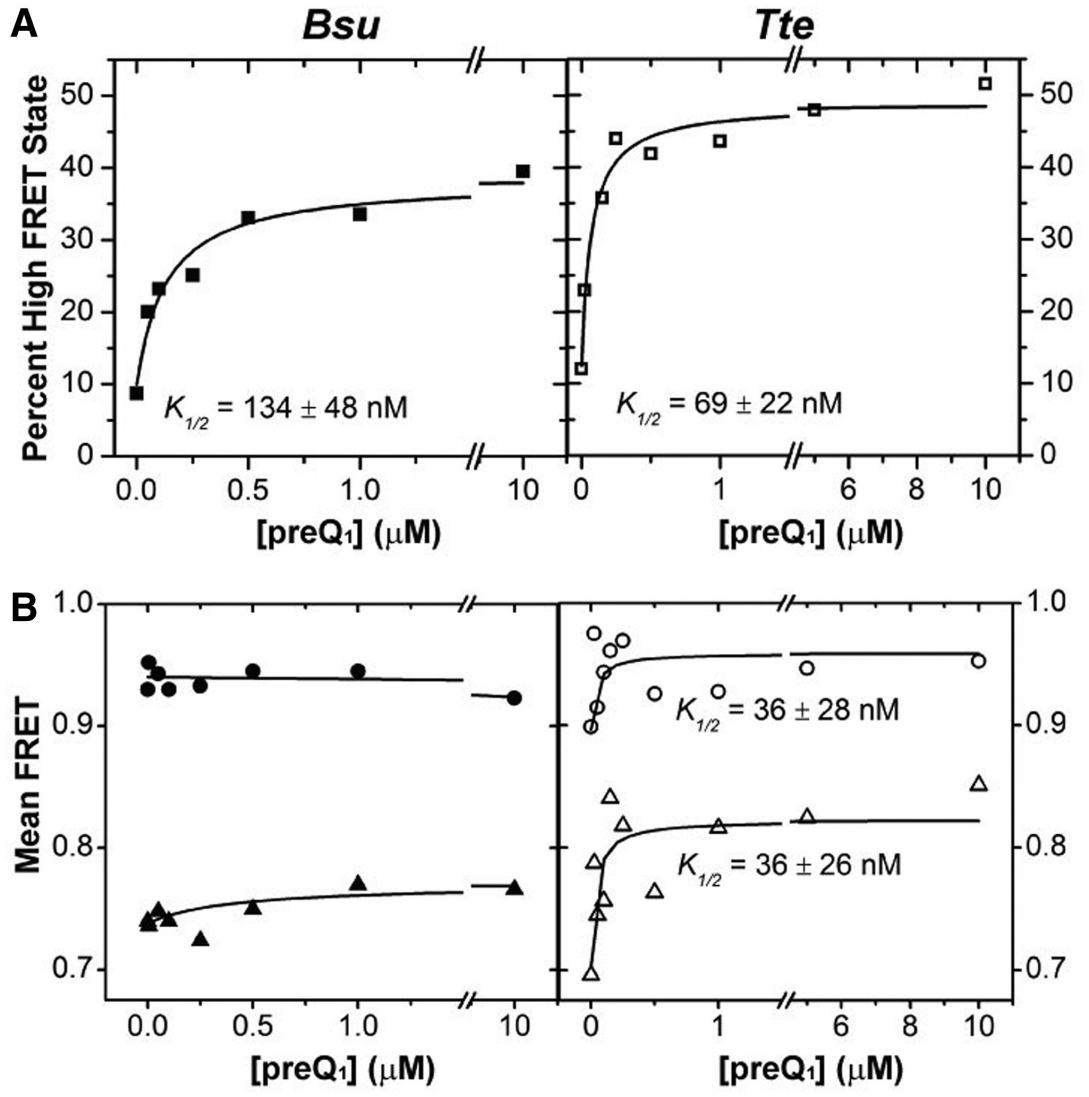
Effect of ligand on the distribution of the mid- and high-FRET states. (**A**) The FRET histograms of Figure 2 were quantified, and the percentage high-FRET state was plotted as a function of ligand concentration. The data were fit with a non-cooperative binding isotherm and the respective apparent *K_1/2_* values are indicated for both *Bsu* (closed symbols) and *Tte* (open symbols). (**B**) The centers of the Gaussian fits for the mid-FRET (green) and high-FRET (blue) states from Figure 2 were plotted as a function of ligand concentration and fit with a non-cooperative binding isotherm, yielding the *K_1/2_* values indicated for the *Tte* riboswitch.

To confirm our preliminary assignments of the mid- and high-FRET states, we studied the effect of ligand on the conformational sampling of the two riboswitches. As the preQ_1_ concentration increases, so does the high-FRET state population, at the expense of that of the mid-FRET state (Figure 2A and B). We fitted each FRET histogram with a sum of two Gaussian functions (Figure 2A and B) and plotted the fraction of the high-FRET state as a function of ligand concentration (Figure 3A). For both riboswitches, the high-FRET population increases with ligand concentration and saturates with half-titration points of K_1/2_ = 134 ± 45 nM and 69 ± 22 nM for the *Bsu* and *Tte* riboswitches, respectively (Figure 3A). To rule out the possibility that the varying number of molecules across our ligand titrations skews our results, we performed an analysis wherein 100 molecules were randomly chosen for each condition, then analyzed as in Figures 2 and 3. We found no difference in the Gaussian distributions and K_1/2_ values outside the stated errors. We note that the apparent preQ_1_ affinity of the two riboswitches differs somewhat from previously reported values [K_1/2_ = 50 nM in 50 mM Tris–HCl (pH 8.3), 20 mM MgCl_2_, 100 mM KCl (32) and 2 nM in 10 mM sodium cacodylate (pH 7.0), 3 mM MgCl_2_ (34) for the *Bsu* and *Tte* riboswitches, respectively]. This may be due to differences in technique (in-line probing and surface immobilization, respectively) and/or buffer conditions, especially pH and Mg^2+^ concentration, which we later show to have a significant effect on the compactness of the *Bsu* riboswitch. Of note, the ligand binding affinity of the *Tte* riboswitch measured here by smFRET and previous work by surface plasmon resonance (SPR) (34) was obtained at room temperature. However, the affinity of the *Tte* riboswitch at physiological growth temperatures of *Thermoanaerobacter tengcongensis* (50–80°C) may be well lower. We therefore used ITC to investigate the effect of temperature on the ligand binding affinity of the *Tte* riboswitch. The K_D_ measured at 25°C is 7.3 ± 2.3 nM (with a reaction stoichiometry N = 0.98), close to the K_D_ measured using SPR (34). At a temperature of 60°C, the ligand binding affinity decreased ∼60-fold with a K_D_ of 430 ± 60 nM (Supplementary Figure S5 and Supplementary Table S3). The lower N value of 0.75 indicates that not all RNA is competent to bind the ligand at 60°C. (Please note that the K_D_ value could not be measured at the optimum *Tte* growth temperature of 75°C due to instability of the instrument). Our ITC data show that, although the affinity is reduced at high temperature, the *Tte* riboswitch still binds ligand with significant affinity.

These observations strongly implicate the high-FRET state as the ligand-bound fully folded state. We note that even at preQ_1_ concentrations as high as 10 µM, however, the fraction of this folded state does not shift above ∼50% for either of the two riboswitches, in part because both riboswitches remain dynamic and sample the pre-folded state, in part perhaps due to the existence of alternative RNA conformations incapable of binding ligand (26,37). In both riboswitches, ligand addition also causes changes in the transition kinetics between the mid- and high-FRET states (Supplementary Figures S2 and S3). Although the *Tte* riboswitch becomes less dynamic with slower transitions, the *Bsu* riboswitch displays increased dynamics with clear two-state transitions. These observations further support the notion that the ligand-free *Bsu* riboswitch undergoes very fast transitions that are slowed down by the ligand through stabilization of the folded state, thus enabling detection at our 100 ms time resolution.

**Figure 4.**
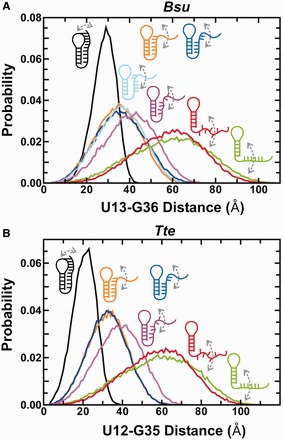
Coarse grained TOPRNA simulations predicting distance distributions between the fluorophore labeled residues as a function of specific interactions in the *Bsu* (**A**) and *Tte* (**B**) riboswitches. Color code is as follows: green, stacked 3′ tail; red, unstacked 3′ tail; purple, blue, orange and cyan (A, only), partially docked into the P1 and/or P2 stem with varying degrees of intersegmental and stacking interactions as indicated; black, fully folded as found in the ligand-bound crystal structures (see Supplementary Tables S1, S2 and S4 for details).

To further evaluate the conformations underlying the mid- and high-FRET states, we performed simulations of both riboswitches with a coarse-grained RNA model we term TOPRNA. TOPRNA uses three pseudo-atoms per nucleotide of an RNA parameterized as a freely rotatable polymer with RNA-consistent bond lengths and angles and with only repulsive non-bonded van der Waals interactions (‘Materials and Methods’ section). Known base-paired regions are parameterized to assume A-form helical structure, and tertiary structures are modeled using dihedral and distance restraints based on the available crystal structures. TOPRNA simulations thus allow us to build a comprehensive picture of the 3D conformational ensemble of the preQ_1_ riboswitches subject to given sets of tertiary structure constraints and inherent space-filling and chain-connectivity properties. Consistent with our initial expectations, we found that ensembles generated without any enforced tertiary restraints possess inter-dye distance distributions that are too long to give a mid-FRET value of ∼0.7 (Figure 4 and Supplementary Table S4). In contrast, the ensembles with either a partially P1- or P2-docked 3′ tail lead to mean donor-acceptor distances in the range of 35–45 Å, highly consistent with that observed for the mid-FRET state (Figure 4 and Supplementary Table S4). We note that these partially folded conformations are still flexible enough to lead to a broad distance (and therefore FRET) distribution as observed for the mid-FRET state (Figure 2A and B). Finally, our simulations of the fully folded ligand-bound state produced a narrower and shorter distance distribution with means around ∼25 Å, as expected for the high-FRET state (Figure 4 and Supplementary Table S4). Altogether, we have strong evidence that the mid-FRET state in both riboswitches consists of a pre-folded conformational ensemble in which the 3′-tail transiently interacts with the minor groove of P1, P2 is partially formed, or a combination of both, whereas the high-FRET state represents a compact less flexible ligand-bound state (or, in the absence of ligand, a folded-like state). We further show that buffer differences, in particular sub-saturating concentrations of Mg^2+^ (Supplementary Figures S6–S8 and Supplementary Results) and low pH, can have significant effects on the pre-folded state of the *Bsu* riboswitch and account for discrepancies with previous studies (26,36). Finally, we find that saturating Mg^2+^ concentrations have a compacting effect on the riboswitches similar to that of high ligand concentrations (Supplementary Figure S8 and Supplementary Results), further highlighting the importance of buffer conditions for RNA folding and tertiary structure.

### smFRET provides evidence that the pre-folded *Tte* riboswitch senses preQ_1_

When fitting the FRET histograms from the preQ_1_ titration in Figure 2 with Gaussian functions, we noticed another difference between the riboswitches. In the case of the *Bsu* riboswitch, the mean FRET values of both the pre-folded and folded states vary little with increasing preQ_1_ concentration (Figure 3B). In contrast, for the *Tte* riboswitch the mean FRET value of particularly the pre-folded state significantly increases on ligand titration, with a K_1/2_ of 36 ± 26 nM (Figure 3B). This observation provides evidence that the *Tte* pre-folded state with a partially formed binding site (without P2 formed yet) ‘senses’ rising preQ_1_ concentrations in that its broad conformational ensemble is increasingly biased toward folded-like (more compact) conformations with increasing ligand concentration, thus narrowing the gap between the mean FRET values of the pre-folded and folded states. The folded state also shifts to higher FRET values, but less so (Figure 3B). There are several ligand-binding mechanisms that could result in these observations. For one, the pre-folded state may rapidly (on the smFRET timescale) sample the native ligand bound state, and the ligand shifts this pre-existing equilibrium by increasing the population of bound conformation, either via conformational selection or induced fit, thus resulting in the observed increased FRET value with increasing ligand concentration. Alternatively, although less likely, ligand binding may stabilize increasingly more native-like pre-folded states of the RNA corresponding to different levels of tail docking. Although our smFRET data themselves cannot resolve the ligand-binding mechanism, they reveal significant differences in the folding behavior of the two riboswitches on the path toward structurally similar ligand-bound end states.

### Gō model simulations reveal tendencies of the *Bsu* and *Tte* riboswitches toward ligand binding by conformational selection and induced fit, respectively

**Figure 5.**
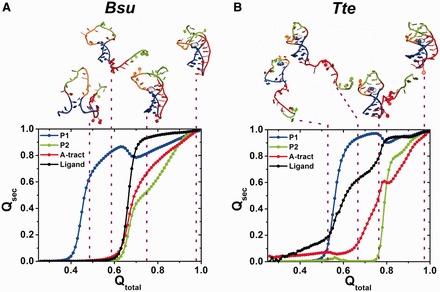
Gō model simulations of single *Bsu* (**A**) and *Tte* (**B**) riboswitch molecules. Fraction of native contacts for each structural component, *Q_sec_* (P1, blue; P2, green; A-tract, red; ligand, black), averaged over each 51 simulations and plotted as a function of the fraction of total contacts observed in the native folded structure, *Q_total_*. Above, characteristic points along the folding pathway are illustrated with each one representative conformation.

To better elucidate the differences in *Bsu* and *Tte* riboswitch folding, we used Gō-model simulations to compare the folding pathways of the *Bsu* and *Tte* riboswitches (Figure 5), essentially as described (25). Gō models are biased toward the fully folded state, leading to smooth folding free energy landscapes and have been used to specifically study the folding pathways of biomolecules including RNA (26,57,58). Starting each simulation from a fully unfolded random conformation, the RNA is allowed to fold to its crystallographically defined native pseudoknot structure (as defined by an appropriate potential function) to probe its most likely folding pathway(s). By performing 51 single molecule simulations for each riboswitch and averaging the fraction of native contacts, Q, as a measure of folding progress, we explored the prevailing folding pathways (Figure 5 and Supplementary Figure S9). We found that both riboswitches follow a similar folding pathway initially as formation of the local P1 stem precedes that of the distal P2 stem. The order of formation of the remaining contacts, however, significantly differs. In the transcriptional *Bsu* riboswitch, ligand binding, stable docking of the 3′-tail and folding of the P2 stem all occur late and almost concomitantly (Figure 5A), whereas in the translational *Tte* riboswitch, ligand binding to the top of P1 occurs early, trailed by 3′-tail docking just before folding of the P2 stem (Figure 5B). Furthermore, C15 of the *Tte* riboswitch (Figure 1C) forms its Watson–Crick base pair with preQ_1_ early in the folding pathway, clearly preceding folding of the remaining binding pocket around the ligand (Supplementary Figure S9B). By contrast, the corresponding C17 of the *Bsu* riboswitch forms its Watson–Crick base pair with preQ_1_ concomitantly with folding of the other binding pocket residues (Supplementary Figure S9A). These divergent tendencies in ligand-mediated folding of the *Bsu* and *Tte* riboswitches essentially recapitulate the classical mechanisms of conformational selection and induced fit, respectively.

### A mutation distal from the binding site impacts ligand binding as predicted by the pre-folded state model

Our data collectively show that the 3′ tail transiently interacts with the P1 stem in the absence of ligand.

**Figure 6.**
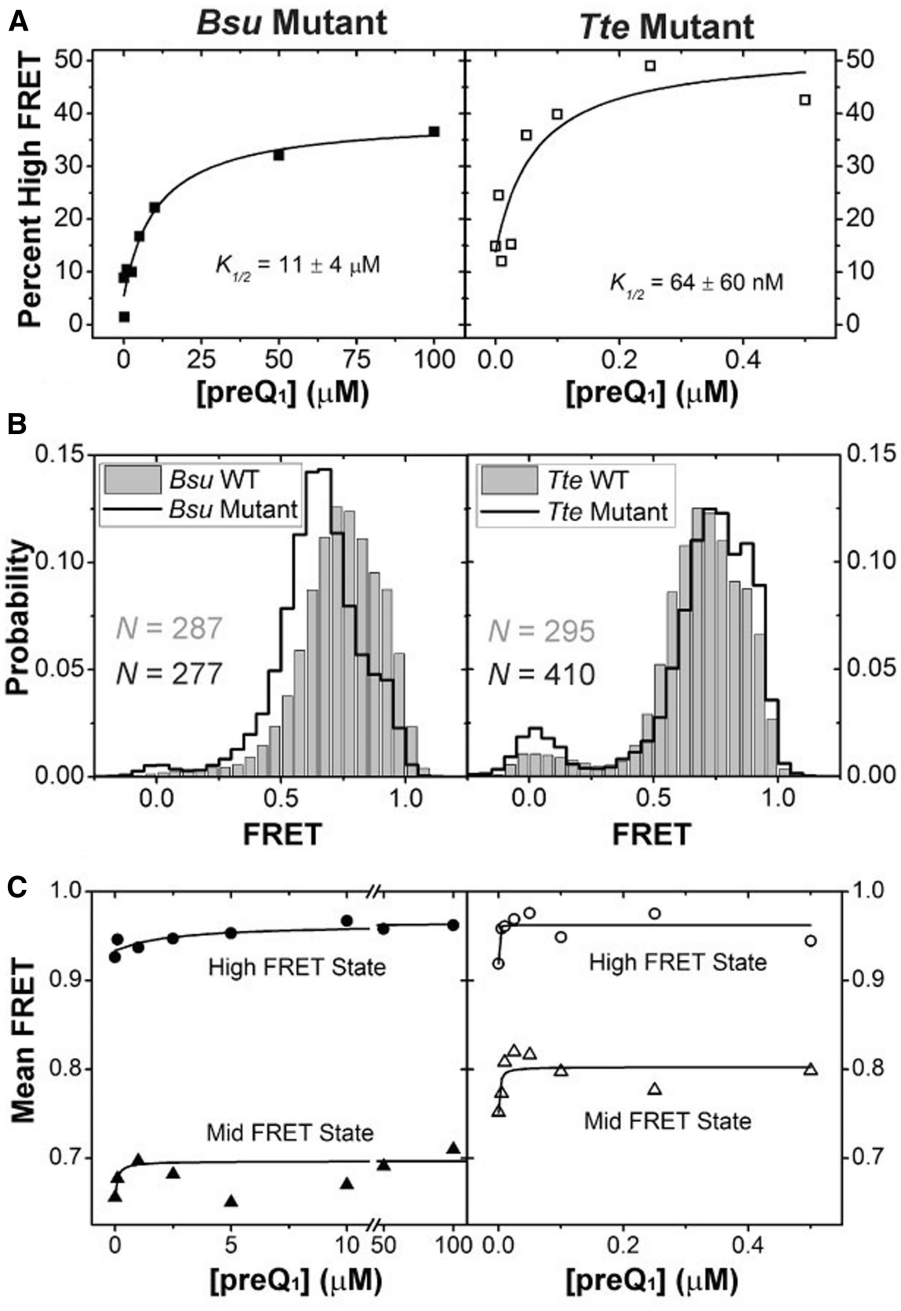
smFRET characterization of riboswitch mutants. (**A**) The Gaussian distributions from Supplementary Figure S10 were quantified and the fraction high-FRET state was plotted as a function of preQ_1_ concentration for the *Bsu* (closed symbols) and *Tte* (open symbols) riboswitches. (**B**) smFRET histograms of the *Bsu* and *Tte* riboswitches in wild-type (WT, gray bars) and mutant (black line) forms, in the absence of preQ_1_. (**C**) The centers of the mid-FRET (triangles) and high-FRET (circles) states in Supplementary Figure S10 were plotted as a function of ligand concentration for both the *Bsu* (closed symbols) and *Tte* (open symbols) riboswitches.

However, the observed shift in mean FRET of the *Tte* mid-FRET state and the lack of such a shift in the *Bsu* riboswitch, coupled with the differences in the Gō-model-derived folding pathways, suggest that the nature of this pre-folded state differs in subtle but potentially important ways. Our previous NMR studies of the isolated 3′ tail of the *Bsu* riboswitch showed that disruption of its contiguous A-tract by an A-to-C mutation resulted in weakened stacking interactions (36). We therefore propose that the uniform A-tract in the 3′ tail of the *Bsu* riboswitch renders it more ordered than the *Tte* riboswitch tail with its C insertion, and thus allows for efficient ligand recognition and binding. This proposal presents a clear testable hypothesis—that one can modulate the ligand binding properties of the preQ_1_ riboswitch by promoting either relative order or disorder of the 3′ tail. To test this hypothesis, we introduced opposite A27C and C25A mutations into the equivalent positions of the *Bsu* and *Tte* riboswitches, respectively. Indeed, disruption of the 3′ tail stacking interactions through the A27C mutation in the *Bsu* riboswitch resulted in a marked decrease in ligand binding affinity by two orders of magnitude relative to wild-type (K_1/2_ = 11 µM versus 134 nM, Figures 3A and 6A and Supplementary Figure S10A), despite the distal nature of the mutation (Figure 1C). In addition, we observed a notable decrease in the mean FRET value of the pre-folded state in the absence of ligand (0.74–0.65, Figure 6B), which only slightly varies across a broad ligand concentration (Figure 6C). This decrease is most likely due to a (partial) loss of 3′-tail rigidity as demonstrated by NMR (36), which our TOPRNA simulations predict will lead to an increase in the inter-fluorophore distance monitored by smFRET (purple line in Figure 4A). By contrast, enhancement of the 3′ tail stacking interactions through the C25A mutation in the *Tte* riboswitch results in no change in binding affinity relative to wild-type (K_1/2_ = 64 nM versus 69 nM, Figures 3A and 6A and Supplementary Figure S10B), accompanied by an increase in the mean FRET value of the pre-folded state (0.75 compared with 0.70 of the wild-type in the absence of ligand, Figure 6B), which only slightly varies across preQ_1_ concentrations (Figure 6C). Consistent with our hypothesis, these results indicate that a distal point mutation in the A-rich 3′ tail of the preQ_1_ riboswitches can change the nature of pre-folded ligand-free conformational ensemble in subtle but powerful ways, affecting not only the ligand binding affinity but also the overall compactness of the pre-folded state.

## DISCUSSION

**Figure 7.**
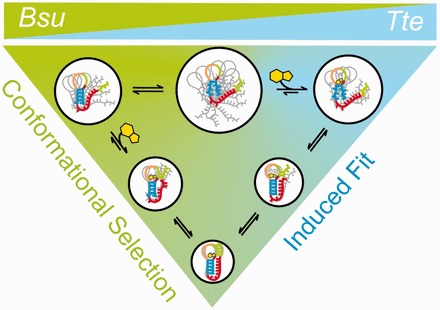
Parsimonious folding model of the *Bsu* and *Tte* preQ_1_ riboswitches. A combination of smFRET and computational simulations support a model in which the preQ_1_ ligand binds late and concomitantly with the docking of the 3′ tail and formation of the P2 stem in the *Bsu* riboswitch, signifying conformational selection (represented in green). By contrast, early binding of the preQ_1_ ligand to a partially unfolded conformation induces folding into the bound structure of the *Tte* riboswitch, consistent with an induced fit model (represented in blue). Both mechanisms are not mutually exclusive, and it is plausible that a combination of both induced fit and conformational selection mechanisms are at work in both riboswitches (59). The size of the white circle and the gray outlines describe the extent of conformational heterogeneity of each state.

Despite acting through completely different modes of gene regulation, the *Bsu* and *Tte* preQ_1_ riboswitches are strikingly similar in their aptamer sequence and structure. By contrast, diverse ensemble-averaging techniques such as NMR spectroscopy, X-ray crystallography and SAXS initially led to distinct structural models for the ligand-free states of the riboswitch, depicting them as either a hairpin with a non-interacting and dynamic 3′ tail (*Bsu*) or as a loose pseudoknot (*Tte*), and left the question of how ligand binding leads to the compact ligand-bound state largely unanswered (26,34). Here, we have used smFRET and both coarse-grained and Gō-model simulations to carry out a detailed side-by-side comparison of the dynamics and ligand-mediated folding of the two riboswitches at the single molecule level. We show that under near-physiological buffer conditions both the ligand-free riboswitches similarly adopt two distinct FRET states—a major, already pre-folded state that, in the case of the *Tte* riboswitch, directly senses ligand, and a minor folded-like state that becomes more populated with increasing ligand concentration. Transitions between the pre-folded and folded states are observed even in the absence of ligand in the *Bsu* riboswitch particularly at 33 ms time resolution. Our coarse-grained simulations suggest that the pre-folded state is an ensemble of conformations with varying degrees of interaction between the (partially) stacked A-rich 3′ tail and the P1-L1 stem-loop. Despite their similarities, smFRET and Gō-model simulations show that the two riboswitches follow, on average, distinct ligand-mediated folding mechanisms, wherein the *Bsu* riboswitch tends to fold more by conformational selection and the *Tte* riboswitch has a relatively greater tendency to fold by induced fit. We also note that both mechanisms appear to be used by both riboswitches, just to differing extents, and that it is difficult to speculate whether one is more advantageous than the other with respect to their overall modes of genetic regulation. Our results support the unifying model in Figure 7, where the differences between the structurally similar transcriptional and translational preQ_1_ riboswitches reduce to subtle, yet significant, relative shifts in their conformational sampling on ligand binding. This model finds further support as we show that remote mutations in the 3′ A-rich tail, which we previously have shown to diminish or enhance stacking of the A’s (36), have significant effects on the ligand binding properties of the riboswitches by shifting their conformational sampling as predicted by the model.

Previous studies of the transcriptional *Bsu* preQ_1_ riboswitch (26) and the closely related *Fnu* (*Fusobacterium nucleatum*) riboswitch (23,24) led to models wherein ligand binding and RNA folding largely occur concurrently, essentially due to a failure to observe interactions between the 3′ tail and the P1-L1 stem-loop in the absence of ligand by NMR. This apparent discrepancy turns out to be due to competing RNA dimerization facilitated by kissing-loop interactions (between U_9_AGCUA_14_ in L1 loop of the *Bsu* riboswitch) at the high RNA concentrations used for NMR, as recent studies attest (29,36), as well as differences in buffer conditions as we show by decreasing the Mg^2+^ concentration and pH (Supplementary Figures S6–S8). The buffer dependence can be rationalized, as 1 mM Mg^2+^ in smFRET experiments is in large stoichiometric excess over the RNA used (10–50 pM during slide binding, which is further lowered as the excess of RNA not bound to the slide is washed away). By comparison, the close-to-millimolar concentration of a 36-nt RNA during standard NMR experiments can render even 10 mM Mg^2+^ sub-stoichiometric relative to the backbone phosphates that need to be charge neutralized to stabilize an RNA’s tertiary structure. In addition, lowering the pH from 7.5 (our near-physiological smFRET buffer) to 6.4 (the standard NMR buffer) likely results in a small population of protonated nucleobases, which is expected to destabilize hydrogen bonding and stacking. Our results are supported by a recent NMR study on the *Fnu* riboswitch that showed that adding Mg^2+^ to the buffer and avoiding dimerization results in a pre-organized pseudoknot-like conformation in the absence of ligand (29). Our observation of a ligand-free ‘pre-folded’ state in the *Bsu* and *Tte* riboswitch aptamers are also supported by recent computational simulations that showed that the 3′-tail interactions with P1-L1 stem-loop are stable in both riboswitches at 300 K even in the absence of ligand (28,30,31).

Our observations are also generally consistent with prior X-ray crystallographic studies of the translational *Tte* riboswitch, which showed only minor differences between the ligand-free (pre-folded) and ligand-bound (folded) states. Complementary SAXS experiments further showed that the ligand-free state additionally opens up in solution compared with the crystal lattice (34). This observation is in accordance with our data that indicate a shift in the mid-FRET state to a more compact structure when increasing the ligand concentration. Here, we have added a dynamic picture for this riboswitch by showing that ligand binding typically occurs early in the RNA folding pathway, albeit with transient interactions of the 3′ tail with the P1-L1 stem-loop already in place, followed by closure of the binding pocket around the ligand through induced fit (Figure 7). By contrast, conformational selection (capture) was proposed for the translational SAM-II riboswitch that also folds into an H-type pseudoknot (60). This conclusion was primarily derived from the slightly (<2-fold) faster ensemble-averaged relaxation kinetics on ligand addition of 2-aminopurine stacking when incorporated into the P2 stem as compared with the P1 stem or 3′ tail. Yet, as ligand binding was only indirectly monitored, it is difficult to establish the exact sequence of ligand binding and P2 stem formation as a way to unambiguously distinguish between conformational selection and induced fit. The authors correctly speculated that multiple pathways may co-exist (60), a notion that we here have expanded on by showing that the structurally related *Bsu* and *Tte* riboswitches are, on average, opposing representatives on a sliding scale of only relative tendencies to fold via ligand-mediated conformational selection and induced fit, respectively (Figure 7).

Our model in Figure 7 is also consistent with the expectation that both transcriptional and translational riboswitches can be poised to bind ligand. Both the *Bsu* and *Tte* riboswitches adopt a prominent dynamic pre-folded conformation in the absence of ligand, with the 3′-tail already pre-positioned, through transient interactions with the P1-L1 stem-loop, close to its eventual placement in the ligand-bound folded state. This poised state is aided by stacking in the A-rich 3′ tail, as evident when we mutate the central A27 to C (*Bsu*), which diminishes the nucleotide’s stacking interactions while keeping its sugar edge intact for hydrogen bonding. Consequently, this mutation dramatically lowers the ligand binding affinity (by ∼80-fold) (Figure 6A). Yet, we find that similar predisposition of the 3′-tail for ligand binding still allows for either conformational selection (*Bsu*) or induced fit (*Tte*). The main distinction between these folding mechanisms lies in the relative longevity of the complex between ligand and the pre-folded conformation with a disordered binding pocket (Figure 7), making the two mechanisms notoriously difficult to distinguish (59). We observe here that the *Tte* riboswitch is characterized by comparably slow, and thus more easily detectable, conformational exchange between the pre-folded and folded states as well as a shift of the pre-folded conformational ensemble toward more compact higher-FRET conformers on ligand encounter. It is tempting to speculate that these experimental distinctions from the *Bsu* riboswitch may provide a general signature for a riboswitch favoring ligand-induced fit. Other signatures are the early ligand binding compared with binding pocket folding, as observed in our Gō-model simulations, and the relative fractional flux through the pathway, which requires a full assessment of all pathway rates that has not yet been experimentally accomplished (59). In general, a combination of both mechanisms is often used in complex biomolecular binding processes, and the major mechanism followed depends on many factors including ligand and RNA concentrations (59). Moreover, even when a ligand primarily selects a specific conformation with a preformed binding pocket, as appears to be the case for the *Bsu* riboswitch, the binding pocket has to still close to entirely envelop the ligand. Caution is therefore warranted when assigning one or the other mechanism to a specific riboswitch.

A previous SPR study on the ligand binding affinity of the *Tte* riboswitch showed a tight binding interaction at 25°C with a K_D_ of 2 nM (34). Our ITC data at 25°C show a comparable affinity (K_D_ = 7.3 nM) when the riboswitch is free in solution, which is similar to the phylogenetically unrelated class II preQ_1_ riboswitch (K_D_ = 17.9 nM) analyzed by ITC under the same conditions. The *Tte* riboswitch loses significant affinity (K_D_ = 425 nM) at temperatures as high as 60°C (Supplementary Figure 5 and Supplementary Table 3), and the affinity is expected to further decrease at the optimum growth temperature of 75°C. Despite this decrease, it still binds preQ_1_ surprisingly well with an affinity comparable with that of the class-I preQ_1_ riboswitch (K_D_ = 283 nM) from *F. nucleatum* at 25°C (24). Interestingly, the *Tte* riboswitch has a shorter L2 loop and lacks 2 nt that are unresolved in the *Bsu* crystal structure (22), suggesting they are flexible. The pre-folded state ensemble of the *Tte* riboswitch thus may require less sampling and be able to fold more efficiently around a transiently bound ligand than the *Bsu* riboswitch to achieve induced fit. Alternatively, this feature may be related to either its function as a translational riboswitch or its origin from a thermophilic, although genetically closely related (61) bacterium. Conversely, the comparably faster transitions of the *Bsu* riboswitch between the pre-folded and folded states may help it rapidly bind ligand by conformational selection within the short time window (<2 s) before the transcribing RNA polymerase clears the downstream expression platform. Single molecule force experiments on the structurally similar *pbuE* and *add* adenine riboswitch aptamers showed that they sample similar conformational ensembles but differ subtly in their folding pathways and dynamics that relate to their distinct functions as transcriptional and translational riboswitches, respectively (62). Given that our work also shows similarities in the conformational distributions of the *Bsu* and *Tte* riboswitches with subtle differences in their dynamics and folding, this appears to be a common feature of structurally similar but functionally different riboswitches. More such comparative studies are needed to understand how riboswitch structure and dynamics are fine-tuned by nature to function through different gene-regulation mechanisms. Given the great impact that we find environmental (buffer) conditions to have, future studies should ideally test such hypotheses under the growth conditions of the bacterium from which a given riboswitch is derived.

In summary, we have used smFRET, NMR and computational simulations to characterize the folding behaviors of two structurally similar but functionally distinct transcriptional and translational riboswitch aptamers. Our work presents direct evidence for a ligand-free pre-folded conformation on the folding pathway of both riboswitches, poised to bind ligand. Furthermore, our data yield evidence that the pre-folded state of the translational riboswitch with only a partially formed binding site directly senses and binds ligand. Our work thus reveals that even small structurally similar RNAs can adopt distinguishable folding mechanisms, consistent with recent observations for highly homologous proteins (63). We also demonstrate the fine-tuned conformational sampling of these riboswitches, as mutation of a single nucleotide distal from the ligand binding pocket has dramatic effects on ligand binding and, therefore, gene regulation. This may be exploited in the future engineering of riboswitches for gene regulatory functions.

## SUPPLEMENTARY DATA

Supplementary Data are available at NAR Online, including [21, 23, 25, 26, 28, 33, 36, 45, 59, 64, 65].

## FUNDING

National Institutes of Health [GM062357 to N.G.W., GM063162 to J.E.W., RR012255 to C.B. and R21 GM096156 to C.B. and H.M.A]. Funding for open access charge: National Institutes of Health.

*Conflict of interest statement*. None declared.

## Supplementary Material

Supplementary Data
