# Supplementary material for: Single transcriptional and translational preQ1 riboswitches adopt similar pre-folded ensembles that follow distinct folding pathways into the same ligand-bound structure
Source: Nucleic Acids Res. 2013 Sep 3;41(22):10462–75. doi: 10.1093/nar/gkt798 (PMC3905878; doi:10.1093/nar/gkt798)
Supplement: Supplementary Data [file supp_41_22_10462__index.html]

Single transcriptional and translational preQ1 riboswitches adopt similar pre-folded ensembles that follow distinct folding pathways into the same ligand-bound structure — Single transcriptional and translational preQ1 riboswitches adopt similar pre-folded ensembles that follow distinct folding pathways into the same ligand-bound structure — Supplementary Data 

# Single transcriptional and translational preQ1 riboswitches adopt similar pre-folded ensembles that follow distinct folding pathways into the same ligand-bound structure

## Supplementary Data

files

**Files in this Data Supplement:**

- Supplementary Data - pdf file
