# Supplementary material for: Single transcriptional and translational preQ1 riboswitches adopt similar pre-folded ensembles that follow distinct folding pathways into the same ligand-bound structure
Source: Nucleic Acids Res. 2013 Sep 3;41(22):10462–75. doi: 10.1093/nar/gkt798 (PMC3905878; doi:10.1093/nar/gkt798)
Supplement: Supplementary Data [file supp_gkt798_nar-00939-r-2013-File009.pdf]

Supplementary Information for:

Single transcriptional and translational $preQ_1$ riboswitches adopt similar pre-folded ensembles that follow distinct folding pathways into the same ligand-bound structure

Krishna C. Suddala[1,2], Arlie J. Rinaldi[2,3], Jun Feng[1,3], Anthony M. Mustoe[1], Catherine D. Eichhorn[4], Joseph A. Liberman[5], Joseph E. Wedekind[5], Hashim M. Al-Hashimi[1,3], Charles L. Brooks III[1,3,6] & Nils G. Walter[2,3,*]

[1]Biophysics, [2]Single Molecule Analysis Group, [3]Department of Chemistry, [4]Program in Chemical Biology, University of Michigan, Ann Arbor, MI 48109, USA, [5]Department of Biochemistry and Biophysics, Center for RNA Biology, University of Rochester School of Medicine and Dentistry, Rochester, NY 14642, USA, [6]Center for Theoretical Biological Physics, University of California San Diego, San Diego, CA 92037, USA

* To whom correspondence should be addressed. Tel: +1 734 615 2060; Fax: +1 734 647 4865; Email: nwalter@umich.edu

Supplementary Results

Differences to previous NMR studies are explained by dimerization and variations in Mg^{2+} concentration and pH

Despite subtle differences in their dynamics, the *Bsu* and *Tte* riboswitches in the absence of ligand exhibit surprisingly similar conformational distributions. These observations contrast with previous suggestions from NMR spectroscopy that the ligand-free *Bsu* riboswitch largely resides in a partially folded, open conformation (25,36), whereas the crystallized ligand-free *Tte* riboswitch is found in a conformation highly similar to that when ligand is bound, which becomes only slightly less compact in solution (33). To resolve this apparent discrepancy, we studied the buffer dependence of the *Bsu* riboswitch using smFRET. We observed that by lowering the Mg^{2+} concentration and pH from those of our near-physiological smFRET buffer to those of a typical NMR buffer (15 mM Na_iPO_4, pH 6.4, 25 mM NaCl, 0.1 mM EDTA), the mid-FRET peak of the pre-folded state decreases significantly to a FRET value of 0.62 ± 0.20 with an increased SD (Supplementary Figure S6). These observations suggest that the tertiary interactions between the 3' tail and P1-L1 stem-loop become less favorable, consistent with previous NMR studies that could not detect them (25,36). Furthermore, when this NMR buffer is supplemented with 2 mM Mg^{2+}, the mid-FRET ensemble shifts back up to 0.69 ± 0.16, close to the smFRET buffer value (Supplementary Figure S6). These observations suggest that the addition of Mg^{2+}, and to an extent the increase to a near-physiological pH, favors a more compact conformational ensemble with transient interactions of the 3' tail with the P1-L1 stem-loop.

Although Mg^{2+} is dispensable for $preQ_1$ binding and recent work found no specific binding sites for Mg^{2+} in the ligand-bound *Bsu* riboswitch (26), its role in the ligand-free state of both the riboswitches remains unclear. One Mg^{2+} ion was found adjacent to the ligand binding pocket of the ligand-free *Tte* structure (33), indicating Mg^{2+} may be important in stabilizing the ligand-free state. To further pinpoint the effect of Mg^{2+} on the ligand-free *Bsu* and *Tte* riboswitches, we performed Mg^{2+} titrations in our smFRET buffer and monitored the resulting conformational populations. In the absence of Mg^{2+}, the smFRET population histogram for the *Bsu* riboswitch exhibits a major broad peak around a FRET value of 0.61 ± 0.20 and a minor peak around 0.92 ± 0.06 (Supplementary Figure S8A). Similarly, the *Tte* riboswitch

shows a FRET peak around 0.71 ± 0.17, accompanied by a peak at 0.94 ± 0.06 (Supplementary Figure S8B). The lower mean FRET value and larger width of the *Bsu* mid-FRET state is consistent with our observations in NMR buffer (Supplementary Figure S6) and shows that the ligand-free *Tte* riboswitch is more compact than the *Bsu* riboswitch in the absence of Mg^{2+}. Increasing the Mg^{2+} concentration at constant ionic strength results in a higher mean FRET value and smaller width of particularly the mid-FRET state for both riboswitches (Supplementary Figure S8), suggesting that this ensemble becomes more compact and ordered. In addition, the relative fraction of the high-FRET state increases, approaching ~34% and ~36% at 10 mM Mg^{2+} for the *Bsu* and *Tte* riboswitches, respectively. These values are similar to the fractions of high-FRET state at high ligand concentrations. We conclude that high concentrations of Mg^{2+} alone, in the absence of ligand, can induce compact folded-like conformations, consistent with recent studies of the SAM-II riboswitch (59,64).

To further reconcile our smFRET data with those from the previous NMR study of the ligand-free *Bsu* riboswitch (25,36), we studied the buffer dependence of the *Bsu* riboswitch using solution-state NMR. Our previous NMR studies of the *Bsu* aptamer revealed a kissing-dimer interaction involving the palindromic L2 loop sequence 5'-U_9AGCUA_{14}-3' (Figure 1C) (36), as observed also for other $preQ_1$ riboswitches (23). A double C12U/C15U mutant eliminates dimer formation at the high concentrations used for NMR (25,36). We observed that, in contrast to the wild-type *Bsu* aptamer, Mg^{2+} addition now causes significant chemical shift perturbations in the absence of ligand; notably, residues around the ligand binding site move to unusual spectral positions that are typically associated with tertiary interactions (Supplementary Figure S7), suggesting that the ligand binding pocket is in a folded-like conformation. Yet some of these new NMR resonances differ from those in the ligand-bound conformation (Supplementary Figure S7), indicating a distinct Mg^{2+}-dependent conformation. In addition, resonances corresponding to the 3' tail interacting with the P1 helix are not observed, indicating that it does not stably dock with the helix even upon addition of Mg^{2+}. These findings agree closely with our smFRET-monitored Mg^{2+} titration (Supplementary Figure S8) and together provide strong evidence that the capacity to form dimers at the high concentration used for NMR as well as buffer differences, mainly Mg^{2+} account for the discrepancies between smFRET and NMR studies of the ligand-free *Bsu* riboswitch. This is also clearly demonstrated in a recent NMR study on the *Fnu* $preQ_1$ riboswitch (28).

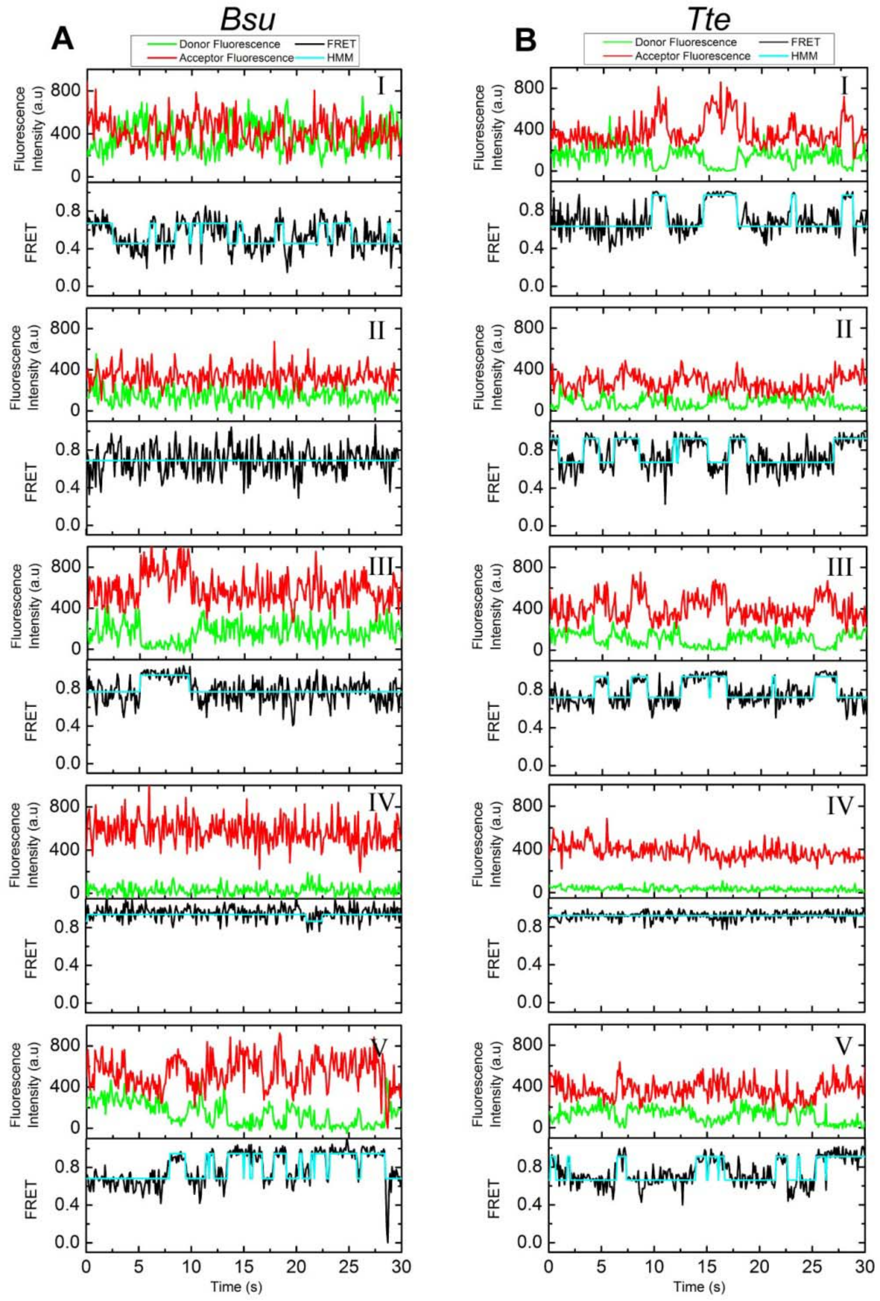

Supplementary Figure S1. Raw smFRET traces of the *Bsu* and *Tte* preQ$_1$ riboswitches in the absence of ligand. (**A**) Five representative time traces illustrating donor (green) and acceptor (red) intensities with corresponding FRET (black) traces for the *Bsu* riboswitch. Computed HMM (cyan) fits are overlaid on the FRET trace. (**B**) Same as **A**, but for the *Tte* riboswitch.

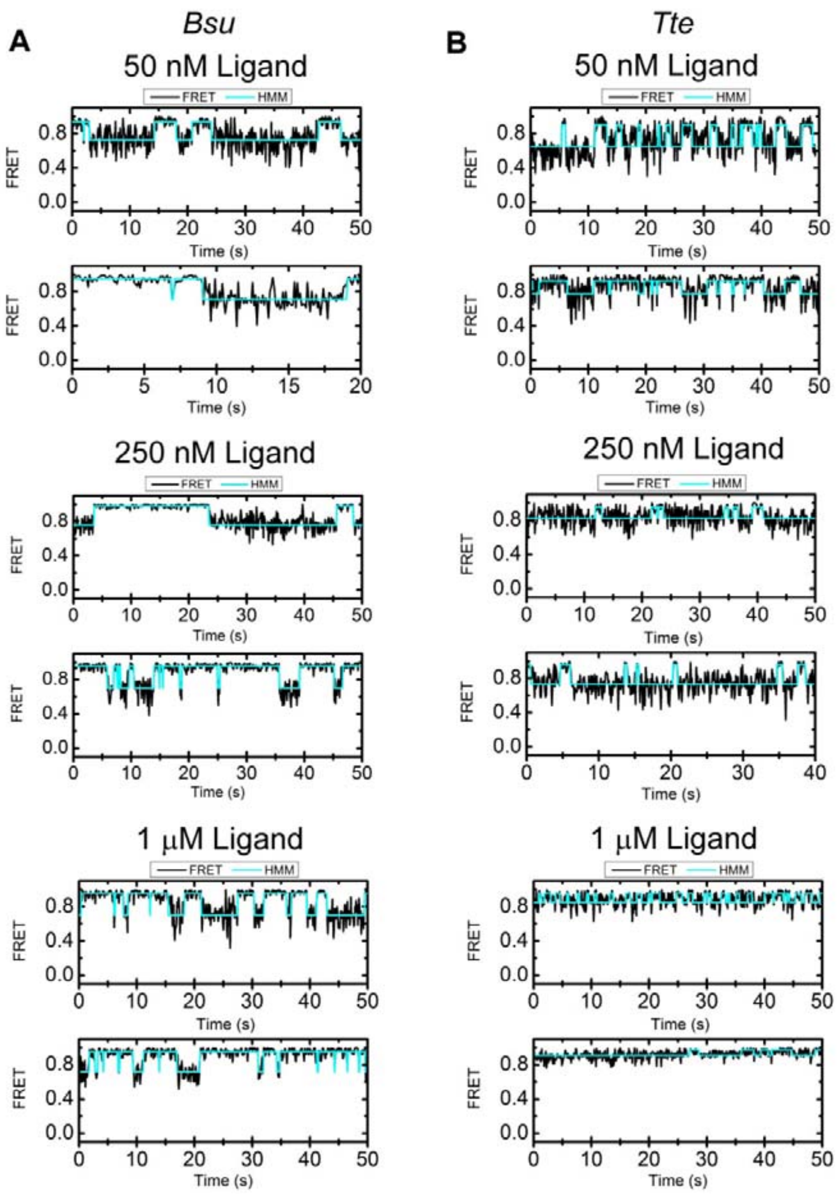

Supplementary Figure S2. Exemplary smFRET traces of the *Bsu* and *Tte* riboswitches in the presence of preQ$_1$ ligand, showing differences in dynamics. (**A**) smFRET traces (black) for the *Bsu* riboswitch with HMM fits (cyan) overlaid. (**B**) Same as **A**, but for the *Tte* riboswitch. The *Bsu* riboswitch shows fewer transitions at low (50 nM) ligand concentration than the *Tte* riboswitch. Conversely, at intermediate (250 nM) and high ligand concentrations (1 μM), the *Tte* riboswitch shows less dynamics than the *Bsu* riboswitch.

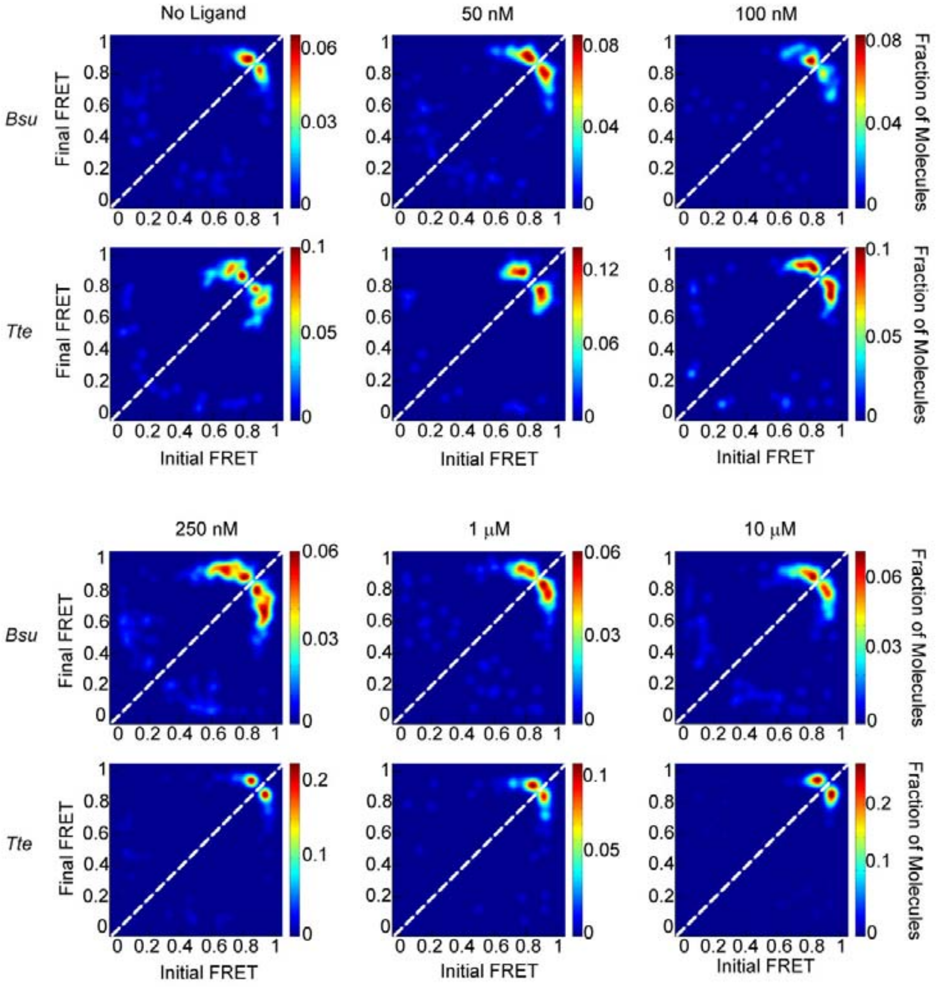

Supplementary Figure S3. Transition occupancy density plots (TODPs) of the preQ$_1$ riboswitches at varying ligand concentrations. TODPs (45) are displayed as heat maps illustrating the fraction of all molecules that exhibit a specific transition from an initial FRET state to a final FRET state for the *Bsu* and *Tte* riboswitches, as indicated. The plots highlight the differences in the transitions between the pre-folded (~0.7 FRET) and folded (~0.9 FRET) states as a function of ligand concentration; transitions between the pre-folded and folded states are seen as off-diagonal contours. In the ligand-free *Tte* riboswitch, these contours move closer to the diagonal (dashed line) upon increasing the ligand concentration, indicative of the pre-folded and folded states becoming structurally more similar. By contrast, for the ligand-free *Bsu* riboswitch, the TODP with no ligand displays contours close to the diagonal that move farther away with increasing ligand concentration (until ~250 nM ligand).

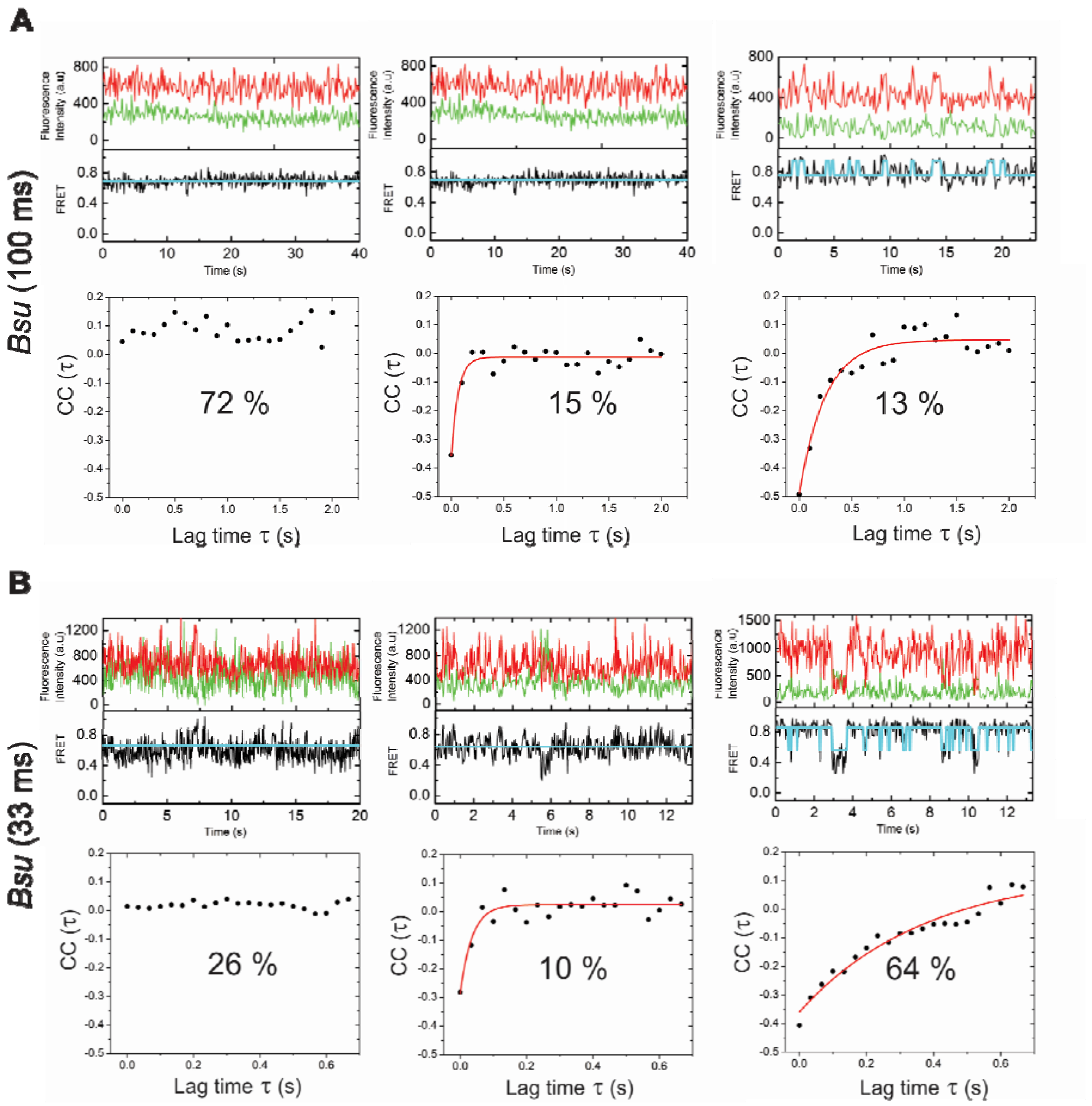

Supplementary Figure S4. Donor-acceptor cross-correlation analysis of exemplary smFRET traces of the *Bsu* riboswitch in the absence of preQ$_1$. (A) Cross-correlation analysis at 100 ms time resolution, showing smFRET traces with their HMM fits (cyan, top panel) and cross-correlation functions, fit with single-exponentials (red, bottom panel). Fractions are given for each of three observed behaviors: left, trace with no detectable dynamics; middle, trace with fast dynamics as shown by cross-correlation between the donor and acceptor signals; right, trace with slow dynamics as identified by the HMM. **(B)** Same as in **A** but at 33 ms time resolution. The time constants (τ) for the single exponential fits are: **A**, 0.065 s and 0.082 s; **B**, 0.035 s and 0.39 s.

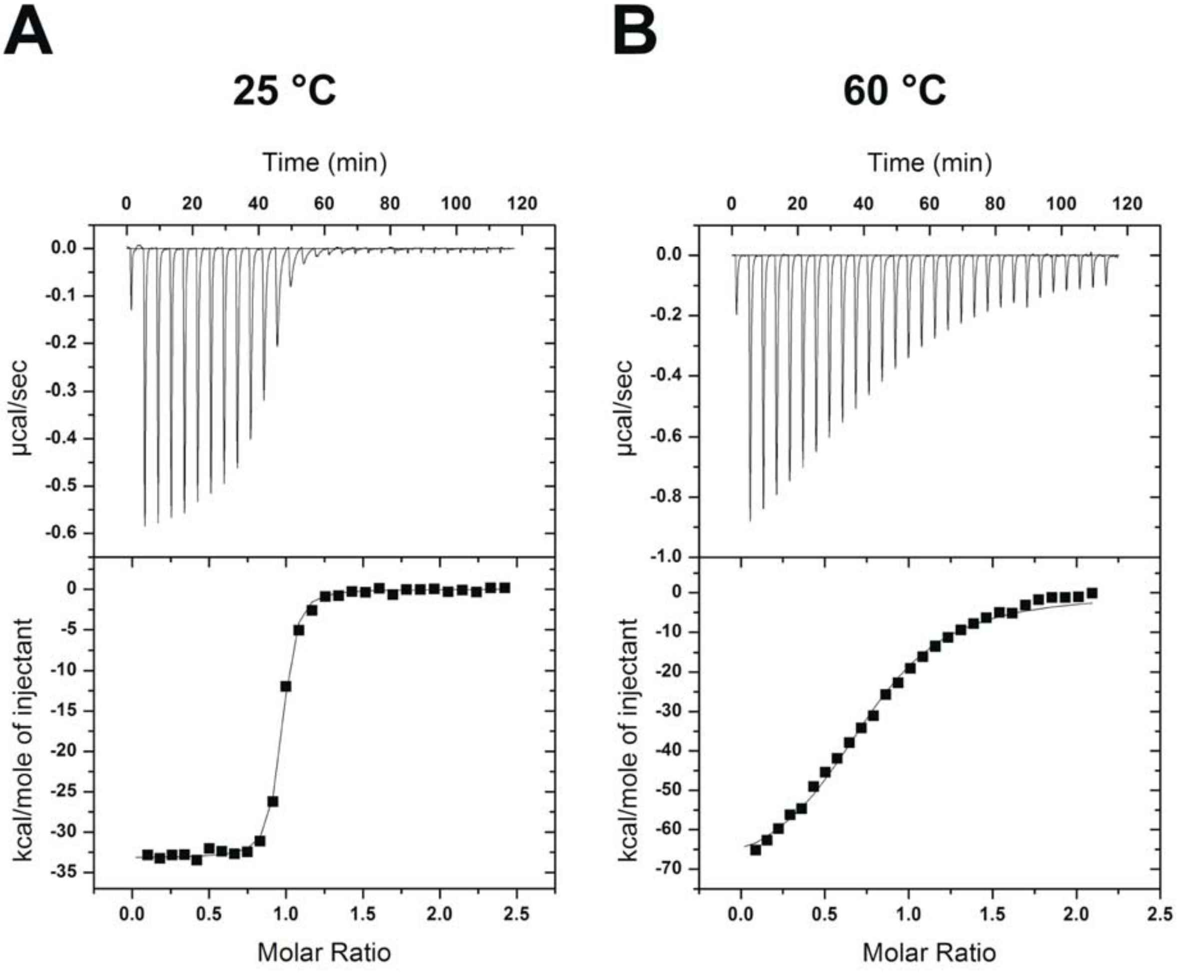

Supplementary Figure S5: Representative isothermal titration calorimetry (ITC) data to measure $\mathbf{preQ_1}$ binding to the *Tte* riboswitch. (A) ITC thermogram of $preQ_1$ binding to the *Tte* riboswitch (top) and resulting binding isotherm fitted with a single-site binding model (bottom) at 25 °C. **(B)** Same as in (A), but at 60 °C.

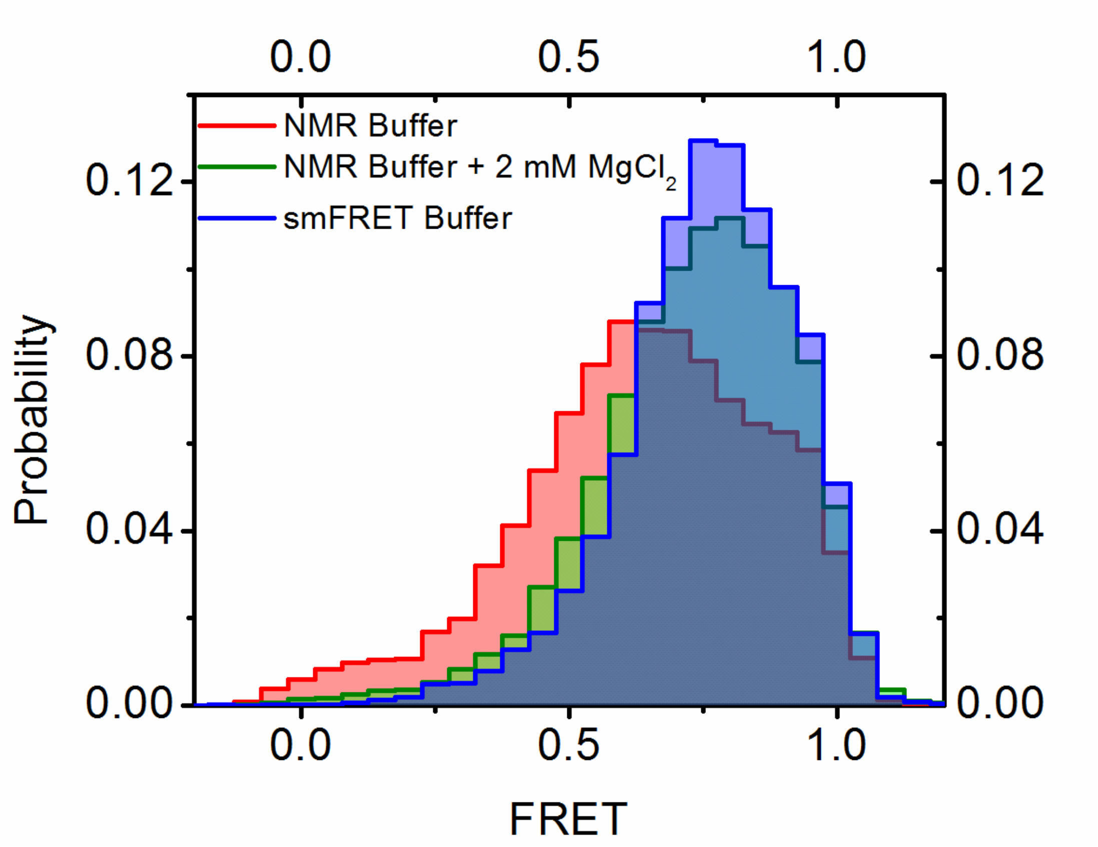

Supplementary Figure S6. Buffer dependence of the ligand-free *Bsu* riboswitch analyzed by smFRET. Low ionic strength NMR buffer (15 mM Na_iPO_4, pH 6.4, 25 mM NaCl, 0.1 mM EDTA; red) shifts the mean FRET value of the mid-FRET state down to 0.61. Supplementing NMR buffer with 2 mM Mg^{2+} (green) shifts the value back up to 0.69, close to the mean FRET value of the mid-FRET state in near-physiological smFRET buffer (50 mM Tris-HCl, pH 7.5, 100 mM KCl, 1 mM $MgCl_2$; blue).

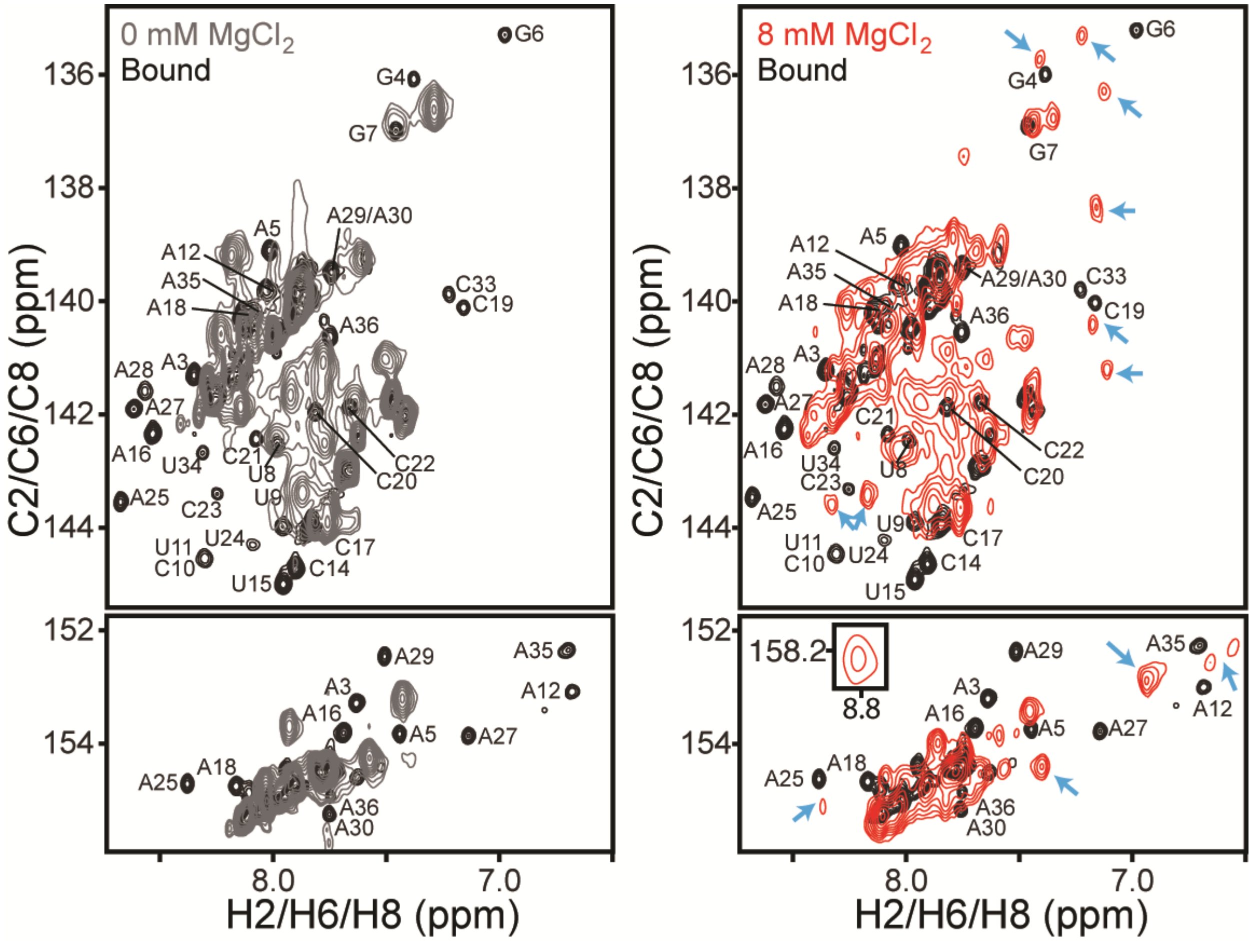

Supplementary Figure S7. NMR characterization of the *Bsu* preQ$_1$ riboswitch – effect of Mg^{2+} on the ligand-free conformation. From left to right: 2D ^1H-^{13}C HSQC comparison of free and preQ$_1$-bound *Bsu* aptamer; comparison of preQ$_1$-free *Bsu* aptamer in the absence of $MgCl_2$ (grey), with 8 mM $MgCl_2$ (red), and with preQ$_1$-bound *Bsu* aptamer in the absence of $MgCl_2$ (black). Addition of $MgCl_2$ gives rise to new peaks that are similar to preQ$_1$-bound chemical shifts in the ligand binding pocket, indicating that Mg^{2+} pre-organizes the ligand-free conformation. Arrows point to chemical shifts indicative of tertiary interactions that are different from those of the ligand bound conformation. Nucleotides are numbered following previous NMR studies on the *Bsu* riboswitch (25,36).

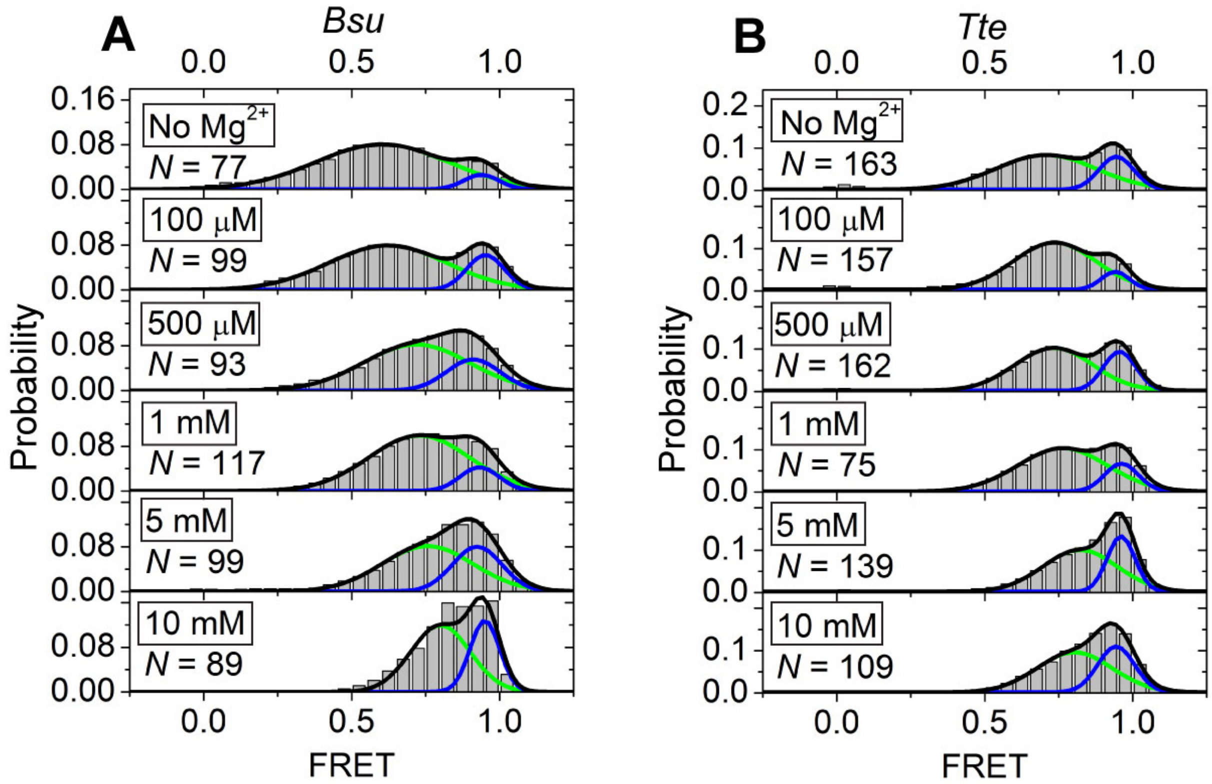

Supplementary Figure S8. Mg^{2+} titration of the *Bsu* (A) and *Tte* (B) riboswitches. The Mg^{2+} concentrations are indicated in the boxes. *N*, number of molecules sampled per condition. Green and blue lines indicate individual Gaussian fits of the mid-FRET and high-FRET states, respectively. Black lines indicate cumulative fits.

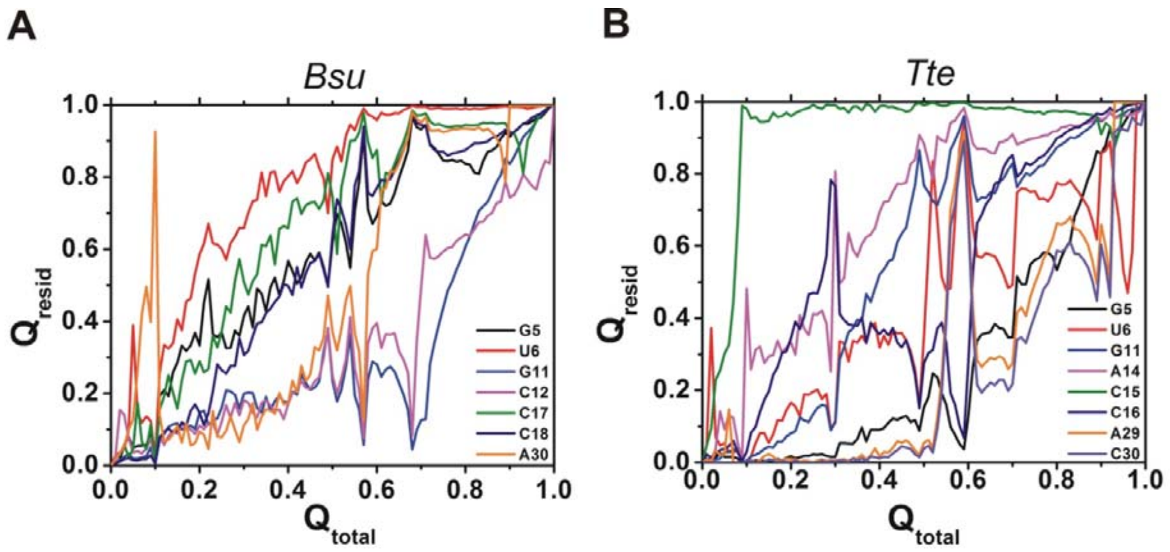

Supplementary Figure S9. Gō model simulations of ligand binding to the *Bsu* (A) and *Tte* (B) riboswitches. The fraction of native contacts, Q_{resid}, formed with $preQ_1$ by each nucleotide, as indicated, is plotted as a function of the fraction of total ligand contacts, Q_{total}. Nucleotides are numbered as in Figure 1C.

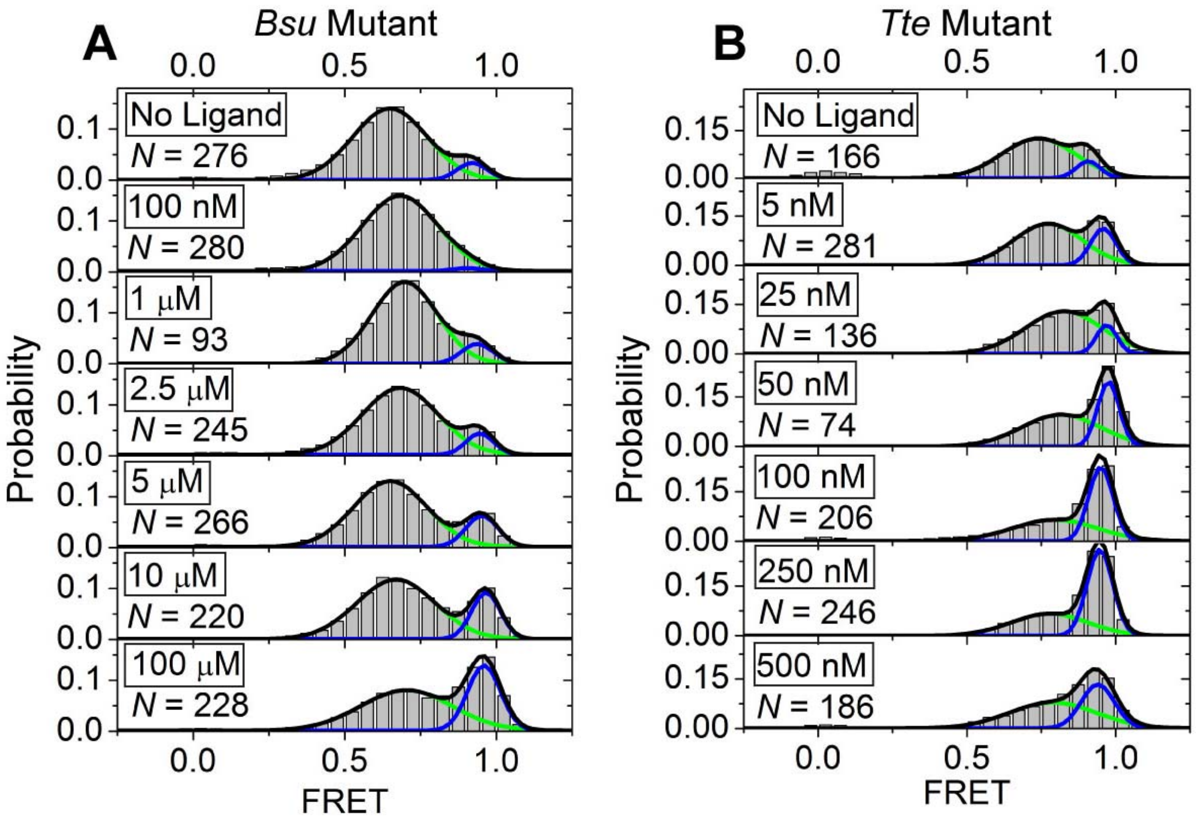

Supplementary Figure S10. smFRET characterization of preQ$_1$ binding to the *Bsu* (A) and *Tte* (B) riboswitch mutants. Ligand concentrations are indicated in the boxes. *N*, number of molecules sampled per condition. Green and blue lines indicate individual Gaussian fits of the mid-FRET and high-FRET states, respectively. Black lines indicate cumulative fits.

Supplementary Table S1. TOPRNA simulation parameters of the *Bsu* preQ$_1$ riboswitch system

Simulation Description	P1 Stem[1]	P2 Stem[2]	Tail Stacking Dihedrals[3]	Tail-P1 Distance Constraints[4]
Unstacked 3' tail (red)	Yes	No	---	---
Stacked 3' tail (green)	Yes	No	A26 to C31	---
Stacked 3' tail with lower tail-P1 interactions (blue)	Yes	No	A26 to C31	U20-A26 (B,S)<br>A3-A26 (B)<br>G4-A27 (B)<br>C19-A27 (B,S)<br>G5-A28 (B,S)<br>C18-A28 (B)
Unstacked 3' tail with lower tail-P1 interactions (purple)	Yes	No	---	U20-A26 (B,S)<br>A3-A26 (B)<br>G4-A27 (B)<br>C19-A27 (B,S)<br>G5-A28 (B,S)<br>C18-A28 (B)
Stacked 3' tail with upper tail-P1 interactions (cyan)	Yes	No	A26 to C31	U6-A29 (B,S)<br>U7-A30 (B,S)
Stacked 3' tail with all tail-P1 interactions (orange)	Yes	No	A26 to C31	U20-A26 (B,S)<br>A3-A26 (B)<br>G4-A27 (B)<br>C19-A27 (B,S)<br>G5-A28 (B,S)<br>C18-A28 (B)<br>U6-A29 (B,S)<br>U7-A30 (B,S)
Ligand-Bound (black)	Yes	Yes	A26 to C31	U20-A26 (B,S)<br>A3-A26 (B)<br>G4-A27 (B)<br>C19-A27 (B,S)<br>G5-A28 (B,S)<br>C18-A28 (B)<br>U6-A29 (B,S)<br>U7-A30 (B,S)

[1]Base pairs A1-U22, G2-C22, A3-U20, G4-C19, and G5-C18 are physically bonded together

[2]Base pairs G11-C31, A10-U32, U9-A33 are physically bonded together, and base pair C8-A34 is enforced through B-atom to B-atom distance constraints.

[3]Backbone dihedral potentials parameterized to enforce A-form helical conformation were added to the residues within the range listed.

[4]Teriary contacts were enforced through the use of flat-well NOE distance constraints with $k_{max}=k_{min}=f_{max}=2.0$ kcal/mol. The constraints were centered on the interaction distances found in chain A of the 3FU2 crystal structure (21) and the well width was set to 1 Å. Letters in parentheses denote whether the constraint used was between two base atoms (B), or between a base and a sugar atom (S), or both (B,S).

Supplementary Table S2. TOPRNA simulation parameters of the *Tte* preQ$_1$ riboswitch system

Simulation Name	P1 Stem Paired[1]	P2 Stem Paired[2]	Tail Stacking Dihedrals[3]	Tail-P1 Distance Constraints[4]
Unstacked 3' Tail (red)	Yes	No	---	---
Stacked 3' Tail (green)	Yes	No	A24 to C30	---
Stacked 3' tail with lower tail-P1 interactions (blue)	Yes	No	A24 to C30	U2-A23 (B,S)<br>A19-A23 (B)<br>G4-A26 (B)<br>C17-A26 (B,S)<br>G5-A27 (B,S)<br>C16-A27 (B)
Unstacked 3' tail with lower tail-P1 interactions (purple)	Yes	No	---	U2-A23 (B,S)<br>A19-A23 (B)<br>G4-A26 (B)<br>C17-A26 (B,S)<br>G5-A27 (B,S)<br>C16-A27 (B)
Stacked 3' tail with all tail-P1 interactions (orange)	Yes	No	A24 to C30	U2-A23 (B,S)<br>A19-A23 (B)<br>G4-A26 (B)<br>C17-A26 (B,S)<br>G5-A27 (B,S)<br>C16-A27 (B)<br>U6-A28 (B,S)
Ligand-Bound	Yes	Yes	A24 to C30	U2-A23 (B,S)<br>A19-A23 (B)<br>G4-A26 (B)<br>C17-A26 (B,S)<br>G5-A27 (B,S)<br>C16-A27 (B)<br>U6-A28 (B,S)<br>A10-A32 (B)

[1]Base pairs C1-G20, U2-A19, G3-C18, G4-C17, and G5-C16 are physically bonded together

[2]Base pairs C9-G33 and G11-C30 are physically bonded together, and non-canonical base pair A10-A32 is enforced through B-atom to B-atom distance constraints.

[3]Backbone dihedral potentials parameterized to enforce A-form helical conformation were added to the residues within the range listed.

[4]Flat-well NOE distance constraints were used as described in Supplementary Table S2. Constraint centers were derived from the 3Q50 crystal structure (33).

Supplementary Table S3. Average Isothermal titration calorimetry (ITC) binding data for the *Tte* riboswitch[a]

Temp (°C)	K_D (nM)	N	ΔH (kcal mol^{-1})	-TΔS (kcal mol^{-1})	ΔG (kcal mol^{-1})
25	7.4 ± 2.3	0.98 ± 0.07	-41.5 ± 11.7	30.3 ± 11.5	-11.2 ± 0.3
60	425 ± 60	0.75 ± 0.01	-97.9 ± 34.7	88.1 ± 34.6	-9.7 ± 0.1

[a]Values represent the average of two independent titration experiments recorded at each temperature (i.e., n = 2).

Supplementary Table S4. FRET values computed from TOPRNA simulated distance distributions

	Bsu				***Tte***			
	R_0=51 Å		R_0=57 Å		R_0=51 Å		R_0=57 Å	
Simulation	d_l=0 Å	d_l=10 Å	d_l=0 Å	d_l=10 Å	d_l=0 Å	d_l=10 Å	d_l=0 Å	d_l=10 Å
Ligand-Bound (black)	0.96	0.82	0.98	0.89	0.99	0.93	0.99	0.96
Stacked 3' tail with all tail-P1 interactions (orange)	0.84	0.65	0.90	0.76	0.87	0.70	0.92	0.79
Stacked 3' tail with lower tail-P1 interactions (blue)	0.81	0.62	0.88	0.73	0.87	0.70	0.92	0.80
Stacked 3' tail with upper tail-P1 interactions (cyan)	0.82	0.62	0.89	0.74	--	--	--	--
Unstacked 3' tail with lower tail-P1 interactions (purple)	0.73	0.52	0.82	0.64	0.77	0.57	0.85	0.68
Stacked 3' tail (green)	0.38	0.24	0.48	0.33	0.39	0.25	0.48	0.34
Unstacked 3' Tail (red)	0.38	0.23	0.49	0.32	0.39	0.24	0.50	0.34

The mean FRET value (E) corresponding to each distance distribution was obtained by assuming complete averaging over the TOPRNA generated ensembles using the following equation (65):

$$E = \left\langle 1 \Big/ 1 + \left(\frac{r + d_l}{R_0} \right)^6 \right\rangle$$

Here, r is the end-to-end distance of a given conformer, R_0 is the approximate Förster radius indicated, and d_l is an additional distance added to estimate possible increases in fluorophore-fluorophore distances that may be expected from the unsimulated linkers.

Supplementary References:

21. Klein, D.J., Edwards, T.E. and Ferre-D'Amare, A.R. (2009) Cocrystal structure of a class I preQ1 riboswitch reveals a pseudoknot recognizing an essential hypermodified nucleobase. *Nat. Struct. Mol. Biol.*, **16**, 343-344.

23. Rieder, U., Kreutz, C. and Micura, R. (2010) Folding of a transcriptionally acting preQ1 riboswitch. *Proc. Natl Acad. Sci. USA*, **107**, 10804-10809.

25. Kang, M., Peterson, R. and Feigon, J. (2009) Structural Insights into riboswitch control of the biosynthesis of queuosine, a modified nucleotide found in the anticodon of tRNA. *Mol. Cell*, **33**, 784-790.

26. Zhang, Q., Kang, M., Peterson, R.D. and Feigon, J. (2011) Comparison of solution and crystal structures of preQ1 riboswitch reveals calcium-induced changes in conformation and dynamics. *J. Am. Chem. Soc.*, **133**, 5190-5193.

28. Santner, T., Rieder, U., Kreutz, C. and Micura, R. (2012) Pseudoknot preorganization of the preQ1 class I riboswitch. *J. Am. Chem. Soc*., **134**, 11928-11931.

33. Jenkins, J.L., Krucinska, J., McCarty, R.M., Bandarian, V. and Wedekind, J.E. (2011) Comparison of a preQ1 riboswitch aptamer in metabolite-bound and free states with implications for gene regulation. *J. Biol. Chem.*, **286**, 24626-24637.

36. Eichhorn, C.D., Feng, J., Suddala, K.C., Walter, N.G., Brooks, C.L., III and Al-Hashimi, H.M. (2012) Unraveling the structural complexity in a single-stranded RNA tail: implications for efficient ligand binding in the prequeuosine riboswitch. *Nucleic Acids Res.*, **40**, 1345-1355.

45. Blanco, M. and Walter, N.G. (2010) Analysis of complex single-molecule FRET time trajectories. *Methods Enzymol.*, **472**, 153-178.

59. Haller, A., Rieder, U., Aigner, M., Blanchard, S.C. and Micura, R. (2011) Conformational capture of the SAM-II riboswitch. *Nat. Chem. Biol.*, **7**, 393-400.

64. Chen, B., Zuo, X., Wang, Y.X. and Dayie, T.K. (2012) Multiple conformations of SAM-II riboswitch detected with SAXS and NMR spectroscopy. *Nucleic Acids Res.*, **40**, 3117-3130.

65. Schuler, B., Lipman, E.A., Steinbach, P.J., Kumke, M. and Eaton, W.A. (2005) Polyproline and the "spectroscopic ruler" revisited with single-molecule fluorescence. *Proc. Natl. Acad. Sci. USA*, **102**, 2754-2759.
